# An isoreticular series of Zr–tricarboxylate based metal–organic nanosheets for water purification applications

**DOI:** 10.1039/d6na00215c

**Published:** 2026-09-09

**Authors:** Jiangtian Tan, Bethany J. Lingard, Ram R. R. Prasad, Louis Kimberley, Kangsheng Bretherton-Liu, Jonathan A. Foster

**Affiliations:** a School of Artificial Intelligence, Nanjing Normal Universit y of Special Education No. 1 Shennong Road, Qixia District Nanjing 210038 China; b School of Mathematical and Physical Sciences, The University of Sheffield Sheffield S3 7HF UK jona.foster@sheffield.ac.uk; c Evove Ltd 7 Shell Green Widnes WA8 0GW UK; d E Hydrogen Ltd 71-75 Shelton Street, Covent Garden London WC2H 9JQ UK

## Abstract

Metal–organic nanosheets (MONs) are porous two-dimensional materials with enormous potential for creating membranes for a wide range of separation applications. Here, we investigate an isoreticular series of Zr–tricarboxylate based MONs bearing different organic ligands for dye separation. A modulated synthesis approach produced monolayer nanosheets with high aspect ratios for all three systems. Composite membranes were formed by spin-coating ethanol suspensions of the nanosheets onto porous polyethersulfone (PES) supports. All three nanosheet systems exhibited consistent performance, with high rejections (72.7–83%) and permeabilities (up to 119.6 L m^−2^ h^−1^ bar^−1^) observed for larger anionic dyes. The observed insensitivity to ligand functionalization, combined with the high permeation of dyes smaller than the pore aperture, indicates that size exclusion is the dominant separation mechanism. This study demonstrates the potential of Zr–tricarboxylate based MONs as a robust and structurally tunable platform for creating high-performance membranes for the effective removal of organic dyes from aqueous environments.

## Abstract 2

Metal–organic nanosheets (MONs) are porous 2D materials ideal for use as membranes in separation applications. Here, an isoreticular series of Zr based MONs prepared on a highly porous substrate are investigated for dye separation applications.

## Graphical abstract

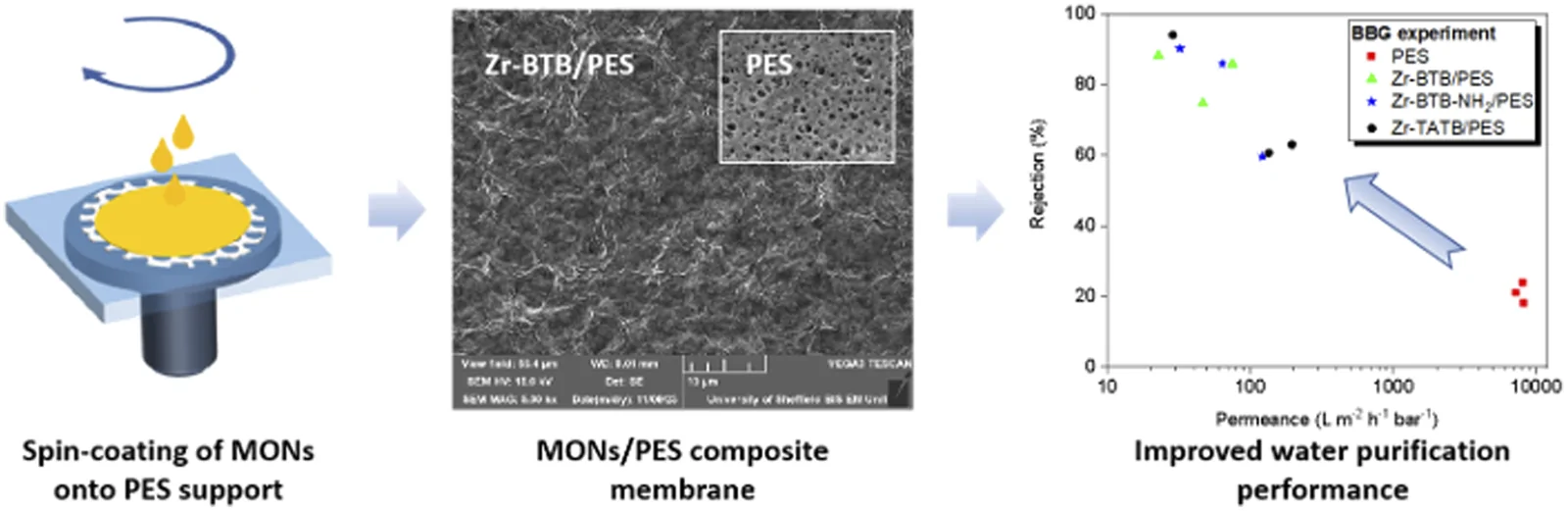

## 1. Introduction

Metal–organic nanosheets (MONs) are a class of porous two-dimensional (2D) materials formed by the co-ordination of metal ions or metal clusters to organic ligands.^1,2^ MONs have diverse chemical properties and tunable structures, making them ideal materials for a wide range of applications, including sensing,^3,4^ catalysis,^5,6^ optoelectronics^7,8^ and drug delivery.^9,10^ In particular, MONs are ideally suited for use in membrane-based separation applications due to their ultrathin nature and well-defined pore sizes.^11–13^ The molecular nature of MONs allows their structures to be systematically tuned to modify pore-size and surface functionalities to enable fine tuning of their properties.

MONs have so far been investigated for a variety of separation applications, including gas separation,^14–16^ oil–water separation,^17^ desalination^18^ and particularly dye separation. For example, Ang and Hong reported membranes formed using polycation-crosslinked zinc porphyrin based MONs that exhibited high water permeance of up to 4243 L m^−2^ h^−1^ bar^−1^ and excellent rejection of organic dyes in aqueous solutions.^19^ Li and co-workers prepared MMMs with BUT-203 (BUT = Beijing University of Technology) MONs and polyethyleneimine by a blending-spin-coating method, which showed a high water permeance of up to 87 L m^−2^ h^−1^ bar^−1^ and high rejection of >97.9% toward anionic dyes with molecular weight larger than 461.^20^ Xuan and co-workers prepared a portable separation device using [Cu_2_(H_4_TTAC)(DMF)_2_]_*n*_ MONs (H_4_TTAC = terphenyl tetracarboxylate acid), which exhibited >99.5% removal of organic dyes phthalocyanine green and copper phthalocyanine.^21^

Amongst the wide variety of MONs reported in the literature, Zr–BTB (BTB = 1,3,5-benzenetribenzoate) has attracted considerable attention due to its high water, chemical and thermal stability and ease of preparation and functionalisation.^22,23^ For example, Zhao and co-workers reported the facile and scalable synthesis of Zr–BTB MONs by a crystal growth modulation method, and the nanosheets they obtained were used to prepare sensors for capacitive gas sensing.^24^ Recently, our group reported that Zr–BTB MONs could be processed into gels in different solvent systems *via* a facile centrifugation process, which could then be used for selective loading and differential release of small molecules based on their size and charge.^25^ The use of Zr–BTB in other applications has also been investigated, including DNA detection,^26^ 2,4-dinitrophenol sensing^27^ and catalysis.^28^ Various methods have also been developed to post-synthetically functionalise Zr–BTB MONs to systematically tune their structures and properties, including functionalisation of Zr–BTB with pillaring ligands^23^ and introduction of indium into Zr–BTB MONs by metal-ion exchange.^29^ In addition, a series of Zr–tricarboxylate based MONs with an isoreticular structure to Zr–BTB have also been reported in the literature, including its amino, methyl and carboxyl derivatives (Zr–BTB–NH_2_, Zr–BTB–CH_3_ (ref. 24) and Zr–BTB–COOH^30^ and Zr–TATB in which the Zr_6_ clusters are linked by 4,4′,4″-s-triazine-2,4,6-triyl-tribenzoic acid (H_3_TATB) ligands forming nanosheets with an average thickness of 1.48 nm.^31^

To our knowledge, only two previous example of Zr–tricarboxylate based MONs for water purification has been reported in the literature, in which Zr–BTB MONs were deposited onto polyacrylonitrile (PAN)-400 supports to prepare composite membranes by a doctor blade casting method.^18^ The Zr–BTB/PAN-400 membranes exhibited high rejection (>95%) of organic dyes, such as acid fuchsin and methyl blue, as well as excellent desalination performance toward Mg^2+^ and Al^3+^ in aqueous solutions. However, compared to other MON-based water purification membranes,^19^ the relatively low water permeances of the Zr–BTB/PAN-400 membranes (1 to 3.2 L m^−2^ h^−1^ bar^−1^) could impede their industrial applications. A recent follow up study explored how the Zr–BTB nanosheets could be post-synthetically functionalised with photo-responsive monodentate carboxylic acids can be used to create subtle variations in interlaying spacing to fine tune permeability (up to 22.7 L m^−2^ h^−1^ bar^−1^).^32^

In this work, we investigate Zr–tricarboxylate based MONs as a promising class of water-stable and structurally tunable two-dimensional materials for membrane-based water purification. In order to explore how ligand functionality influences membrane structure and performance, a series of three isoreticular frameworks were investigated, specifically (Fig. 1a): 1,3,5-benzenetribenzoic acid (H_3_BTB), 2′-amino-5′-(4-carboxyphenyl)-[1,1′:3′,1″-terphenyl]-4,4″-dicarboxylic acid (H_3_BTB-NH_2_) and 4,4′,4″-s-triazine-2,4,6-triyl-tribenzoic acid (H_3_TATB). Ethanol suspensions of the three MONs were spin-coated onto highly permeable polyethersulfone (PES) supports to prepare composite membranes, providing a membrane architecture with substantially higher intrinsic permeability than previously reported PAN-supported systems. The water purification performance of the resulting membranes were systematically studied for a series of dyes with different sizes and charges.

**Fig. 1:**
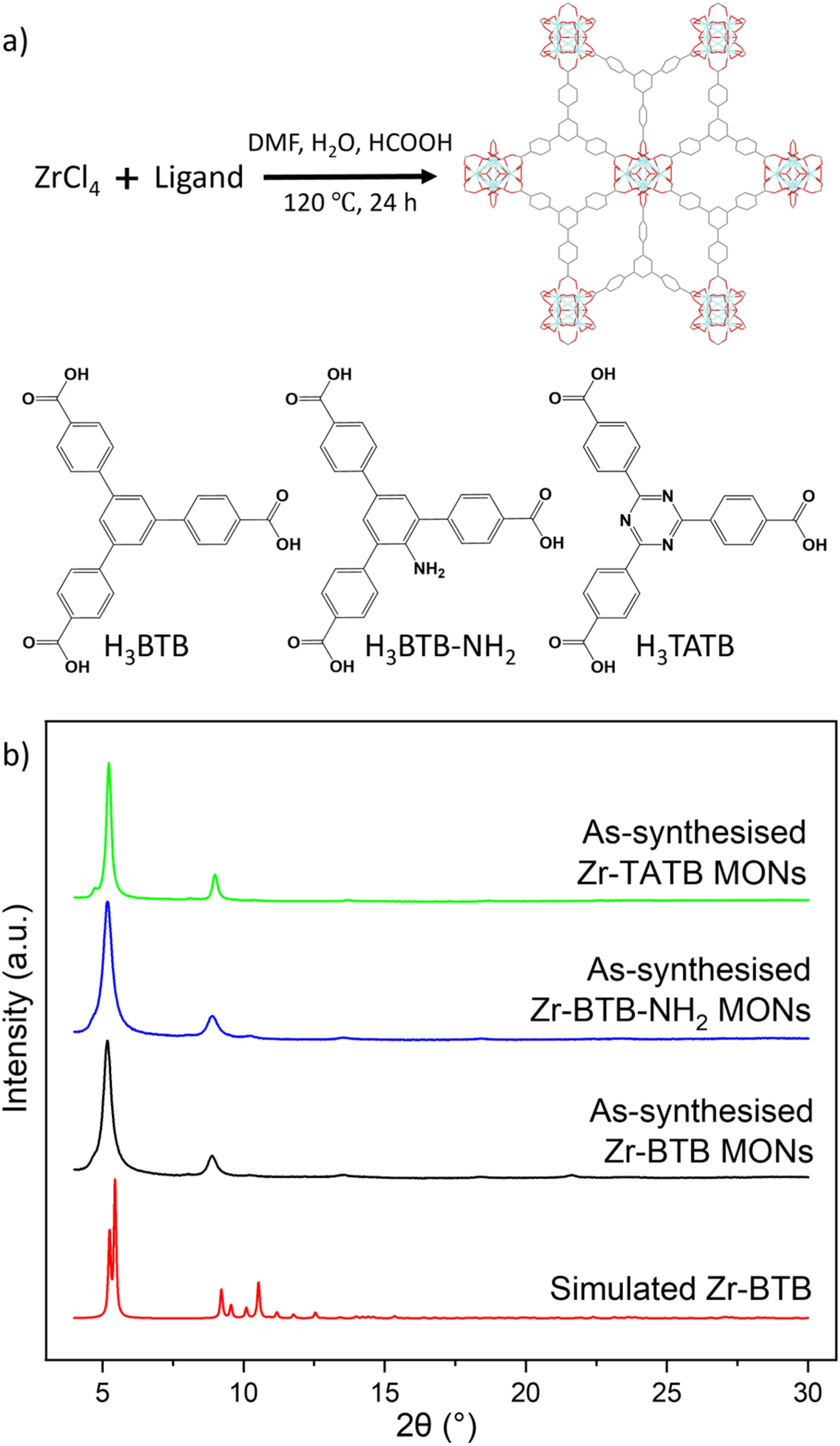
(a) Synthesis of an isoreticular series of Zr–tricarboxylate based MONs using different ligands, including H_3_BTB, H_3_BTB–NH_2_ and H_3_TATB. The crystal structure is downloaded from CCDC (deposition number: 1567188). (b) PXRD patterns of the as-synthesised Zr–BTB, Zr–BTB–NH_2_ and Zr–TATB MONs, comparing with the simulated pattern of Zr–BTB.

## 2. Results and discussion

### 2.1. Preparation and characterisation of MONs

Three Zr–tricarboxylate based MONs were synthesised by adapting the method reported by Zhao and co-workers.^24^ As shown in Fig. 1a, zirconium(iv) chloride (ZrCl_4_, 0.129 mmol) and the tritopic carboxylic acid ligands (H_3_BTB, H_3_BTB–NH_2_ or H_3_TATB, 0.068 mmol) were dissolved in a mixture of formic acid (HCOOH, 2 mL), deionised water (H_2_O, 2 mL) and *N*,*N*-dimethylformamide (DMF, 15 mL), which was then heated at 120 °C for 24 h in a reaction oven. The resulting viscous suspensions of the three MONs were then centrifuged and washed three times with fresh DMF then ethanol. The washed MONs were stored in ethanol as homogeneous suspensions with a concentration of 2.5 mg mL^−1^ until needed.

Ethanol suspensions of the Zr–BTB, Zr–BTB–NH_2_ and Zr–TATB MONs (1 mL) were dried using a hot plate at 80 °C to remove most of the solvent and then at 120 °C for complete drying. The resulting solids were characterised by powder X-ray diffraction (PXRD). The PXRD patterns of the three as-synthesised MONs matched well with the ones reported in the literature,^24,31^ suggesting that phase pure MONs were synthesised (Fig. 1b). Compared to the simulated PXRD pattern of 3D Zr–BTB, the PXRD patterns of the three as-synthesised MONs showed only two peaks at 2*θ* = 5.1° and 8.9°, and the other peaks were not observed. This could be attributed to the extreme aspect ratios of the nanosheets resulting in line broadening and preferred orientation effects.^14,31^ Zr–BTB–NH_2_ and Zr–TATB showed the same characteristic as Zr–BTB, indicating that the three MONs have an isoreticular structure.

The as-synthesised MONs were also characterised using atomic force microscopy (AFM) and the results showed that the three nanosheets had a similar thickness between 1–1.5 nm (Fig. 2a–c). This is comparable to the van der Waals size of Zr_6_ clusters (1.2 nm) in MONs reported in the literature,^33^ indicating the formation of monolayer nanosheets. SEM characterisations of the three MONs confirmed their two-dimensional sheet-like morphologies (Fig. 3a–c and S1–S3) and dynamic light scattering (DLS) results showed that the three MONs have similar particle size distributions in the range of 2–6.5 µm (Fig. S4–S6).

**Fig. 2:**
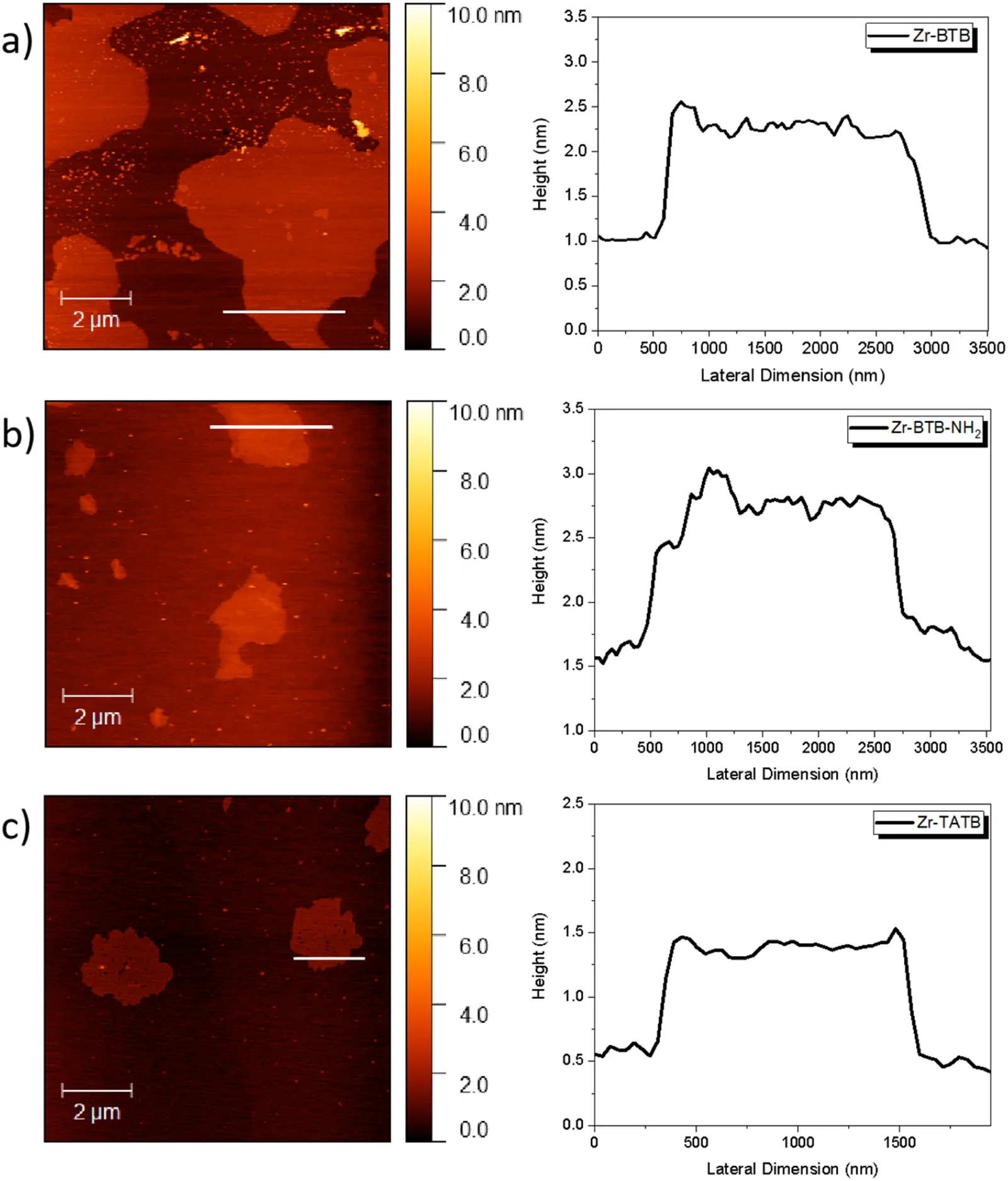
AFM images and corresponding height profiles of (a) Zr–BTB, (b) Zr–BTB–NH_2_ and (c) Zr–TATB MONs.

**Fig. 3:**
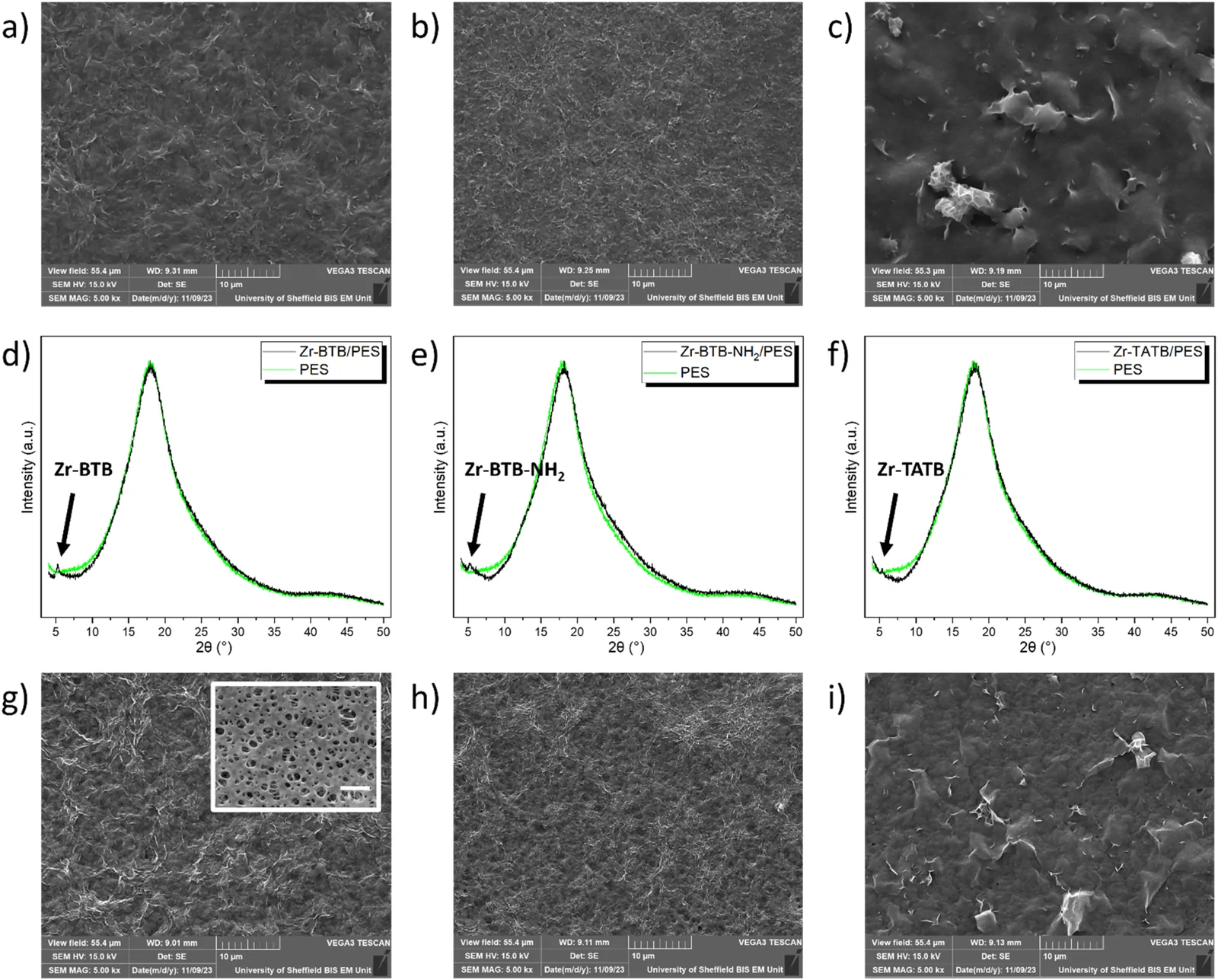
SEM images of (a) Zr–BTB, (b) Zr–BTB–NH_2_ and (c) Zr–TATB MONs. (d–f) PXRD characterisations of the PES support, Zr–BTB/PES, Zr–BTB–NH_2_/PES and Zr–TATB/PES membranes. SEM images of (g) Zr–BTB/PES, (h) Zr–BTB–NH_2_/PES and (i) Zr–TATB/PES membranes. The inset SEM image in (g) shows the PES support, scale bar: 5 µm.

These results indicated that an isoreticular series of Zr–tricarboxylate based MONs with monolayer thickness and similar particle size distributions were successfully prepared by simply varying the organic ligands in the synthesis of Zr–BTB. The reported pore size of Zr–BTB and Zr–BTB–NH_2_ MONs in the literature is 0.54 nm,^15^ smaller than the sizes of organic dyes in aqueous solutions, such as brilliant blue G (BBG, 1.72 × 2.22 × 0.56 nm), methyl (MO, 1.47 × 0.43 × 0.25 nm) and methylene blue (MnB, 1.46 × 0.50 × 0.18 nm, Table 1).^19^ Therefore, all three MONs were considered promising materials for the preparation of water purification membranes for use in dye separations.

**Table 1:** Structures and properties of BBG, MO and MnB dyes and water purification performance of membranes

Dye	Structure	Properties	Membrane	Water permeance (L m^−2^ h^−1^ bar^−1^)	Rejection (%)	Adsorption (%)
BBG	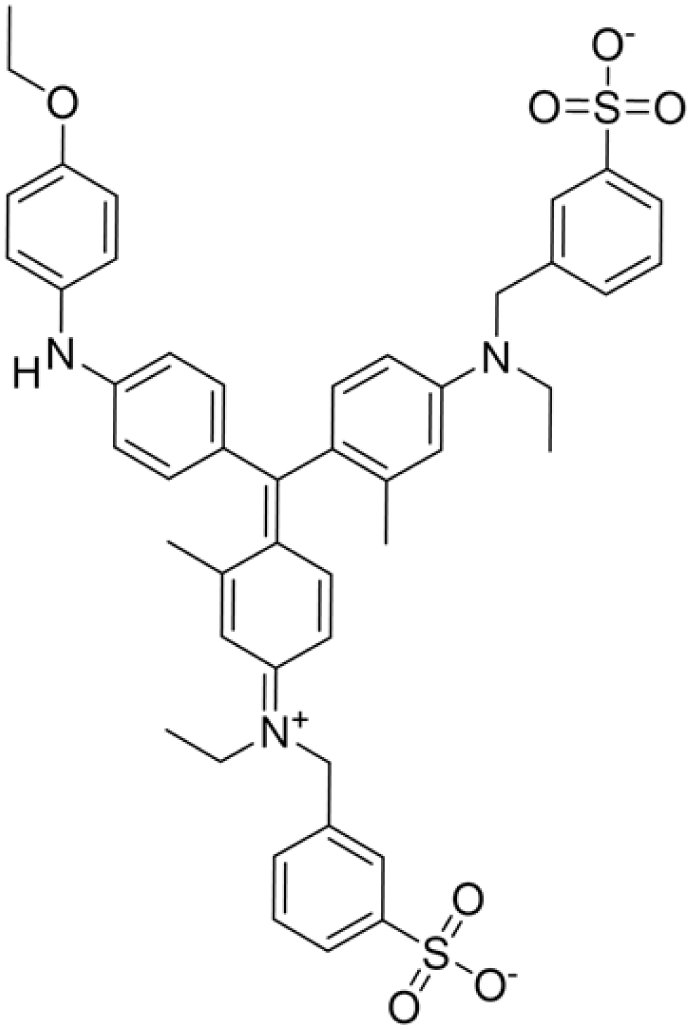	Molecular weight (g mol^−1^) 831.03	PES	7821.7 ± 481	21 ± 3	18 ± 3.6
			Zr–BTB/PES	48.2 ± 26.2	83 ± 7	17.3 ± 9.6
		Dimensions of hydrated BBG^a^ (nm) 1.72 × 2.22 × 0.56				
			Zr–BTB–NH_2_/PES	72.9 ± 45.6	78.7 ± 16.3	21.3 ± 9.5
		Formal charge −1	Zr–TATB/PES	119.6 ± 84.3	72.7 ± 18.5	23 ± 7.8
MO	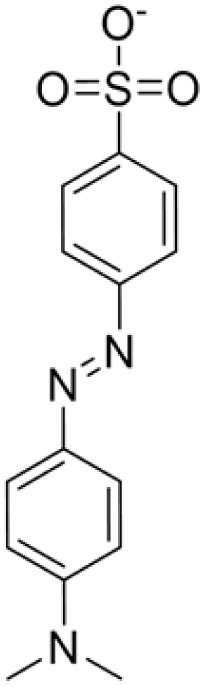	Molecular weight (g mol^−1^) 304.34	PES	9276 ± 515.8	0.9 ± 0.2	0.8 ± 0.2
			Zr–BTB/PES	4599.7 ± 1253.8	1.8 ± 0.4	1.6 ± 0.4
		Dimensions of hydrated MO^a^ (nm) 1.47 × 0.43 × 0.25				
			Zr–BTB–NH_2_/PES	3332 ± 1516	1.4 ± 0.3	1.1 ± 0.2
		Formal charge −1	Zr–TATB/PES	3674.7 ± 1854	1.6 ± 0.6	1.3 ± 0.4
MnB	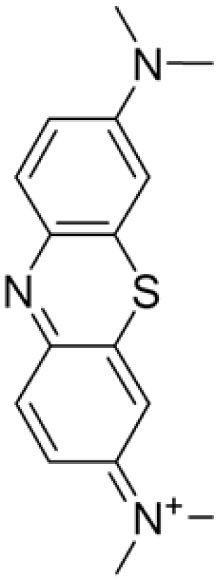	Molecular weight (g mol^−1^) 284.40	PES	9705 ± 393.8	6.9 ± 5.3	6.2 ± 4.8
			Zr–BTB/PES	4769.3 ± 1151.8	25.8 ± 20.4	21.3 ± 16.6
		Dimensions of hydrated MnB^a^ (nm) 1.46 × 0.50 × 0.18				
			Zr–BTB–NH_2_/PES	2194.7 ± 1436.9	17.7 ± 15.6	14.8 ± 13.8
		Formal charge +1	Zr–TATB/PES	2906.7 ± 2193.9	21.3 ± 7.1	18 ± 6.2

a Dimensions of hydrated BBG, MO and MnB dyes were reported by Ang and Hong.^19^

### 2.2. Water purification membranes

Composite membranes of Zr–BTB, Zr–BTB–NH_2_ and Zr–TATB MONs for water purification experiments were prepared by a facile spin-coating method onto highly permeable polyethersulfone (PES) supports with a large pore size of 200 nm. The PES support was fixed to a spin coater and spun at 2500 rpm. Two 500 µL portions of ethanol suspension of MONs (2.5 mg mL^−1^) were deposited onto the PES support and spun for 640 s. The obtained composite membranes (denoted as Zr–BTB/PES, Zr–BTB–NH_2_/PES and Zr–TATB/PES) were dried at room temperature and characterised by PXRD. The PES support is amorphous in nature and showed only one broad peak at 2*θ* = 18° which was found in the PXRD patterns of all three composite membranes (Fig. 3d–f). A small, but characteristic peak belonging to the MONs (at 2*θ* = 5.1°) was observed in the PXRD patterns of the three composite membranes (Fig. 3d–f), indicating that the crystallinity of all three MONs was maintained during membrane preparation.

SEM imaging of the Zr–BTB/PES membrane showed that the deposited MONs formed a continuous layer and confirmed a good adhesion between the MONs and the PES supports (Fig. 3g, S10 and S24). X-ray spectroscopy (EDS) elemental mapping characterisations showed homogeneous distributions of C, O and S elements in the PES support (Fig. S8). Samples prepared with lower loadings of MONs (1.25 mg mL^−1^) showed partial coverage with exposed PES supports whilst higher loadings (5 mg mL^−1^) showed thicker membranes with some cracking (SI Fig. S25). The Zr–BTB–NH_2_/PES membrane (2.5 mg mL^−1^, Fig. 3h and S11) showed a similar surface morphology to the Zr–BTB/PES membrane and EDS elemental mapping characterisations of the two membranes showed the presence of Zr element in them (Zr–BTB/PES: 6.6 wt%, Zr–BTB–NH_2_/PES: 5.4 wt%, Fig. S11 and S13). SEM imaging of the Zr–TATB/PES membrane (2.5 mg mL^−1^, Fig. 3i and S14) showed that it had a similar surface morphology to the other two composite membranes.

Post-filtration PXRD characterisations of all three membranes confirm that the characteristic MON crystallinity is retained after BBG filtration (Fig. S26), indicating no structural degradation of the selective layer. Furthermore, a long-term stability test was conducted on the Zr–BTB/PES membrane by filtering 450 mL of BBG feed—nearly double the standard 250 mL test volume—with permeate collected in 50 mL intervals (Fig. S27 and S28). The membrane exhibited stable water permeance after the initial 50 mL, while rejection progressively increased and stabilised above 88%, yielding an overall rejection of 87% that falls within the experimental error of our standard tests (83 ± 7%). Together with the post-filtration SEM images (Fig. S18–S20), these data collectively confirm that the membranes preserve their morphology, crystalline structure and separation performance under extended operation. These results indicated that the Zr–BTB/PES, Zr–BTB–NH_2_/PES and Zr–TATB/PES membranes with maintained MON crystallinity and similar surface morphologies were successfully prepared, and the use of these membranes in water purification were studied.

### 2.3. Water purification experiments

The water purification performance of the three composite membranes (Zr–BTB/PES, Zr–BTB–NH_2_/PES and Zr–TATB/PES) and the PES supports were investigated for a series of dyes with different sizes and charges (Tables 1 and S2). In addition, the adsorptions of BBG, MO and MnB dyes on the three composite membranes and the PES supports during water purification experiments were also investigated to understand the interactions between the membranes and the dyes with different charges and their effects on the water purification performance of the membranes.

The water purification experiments were conducted using a Sterlitech HP4750 stirred cell operated at 1 bar pressure (Fig. S16) and all experiments were stirred at 500 rpm in order to minimise the concentration polarisation effect.^19^ 0.25 L of aqueous dye solution feed (10 mg L^−1^) was added and the experiment stopped when approximately 0.2 L of permeate was collected. The water purification experiment was repeated three times for each nanosheet system using independently prepared membranes. The water permeance, percentage rejection and percentage adsorption of the membranes for aqueous BBG, MO and MnB dye solutions were calculated as detailed in the experimental section and the results were summarised in Table 1.

The water purification performance of the PES membranes toward negatively charged BBG dyes was studied. As shown in Table 1, the PES supports showed water permeances of 7821.7 ± 481 L m^−2^ h^−1^ bar^−1^ and rejected 21 ± 3% BBG. The base-line variation in these numbers is relatively high, presumably due to batch-to-batch differences in porosity and pore size distribution in these commercially available support membranes as well as challenges in accurately measuring very high permeances.

Spin-coating of Zr–BTB, Zr–BTB–NH_2_ and Zr–TATB MONs onto the PES supports significantly increased their percentage rejection of BBG. In three repeated experiments, the Zr–BTB/PES, Zr–BTB–NH_2_/PES and Zr–TATB/PES membranes exhibited rejection percentages of 83 ± 7%, 78.7 ± 16.3%, and 72.7 ± 18.5% of BBG, respectively (Table 1). The adsorption of BBG on the composite membranes was found to be significantly lower than their rejection of BBG (Fig. 4a and d). This indicated that the high degree of rejection shown by the composite membranes toward the large BBG dye was predominantly due to size exclusion effects by the MONs rather than the adsorption of dyes by the membranes. Significantly, although deposition of the three MONs onto the PES supports decreased their permeances toward aqueous BBG solutions, the membranes prepared in this work retained high water permeances of 48.2–119.6 L m^−2^ h^−1^ bar^−1^.

**Fig. 4:**
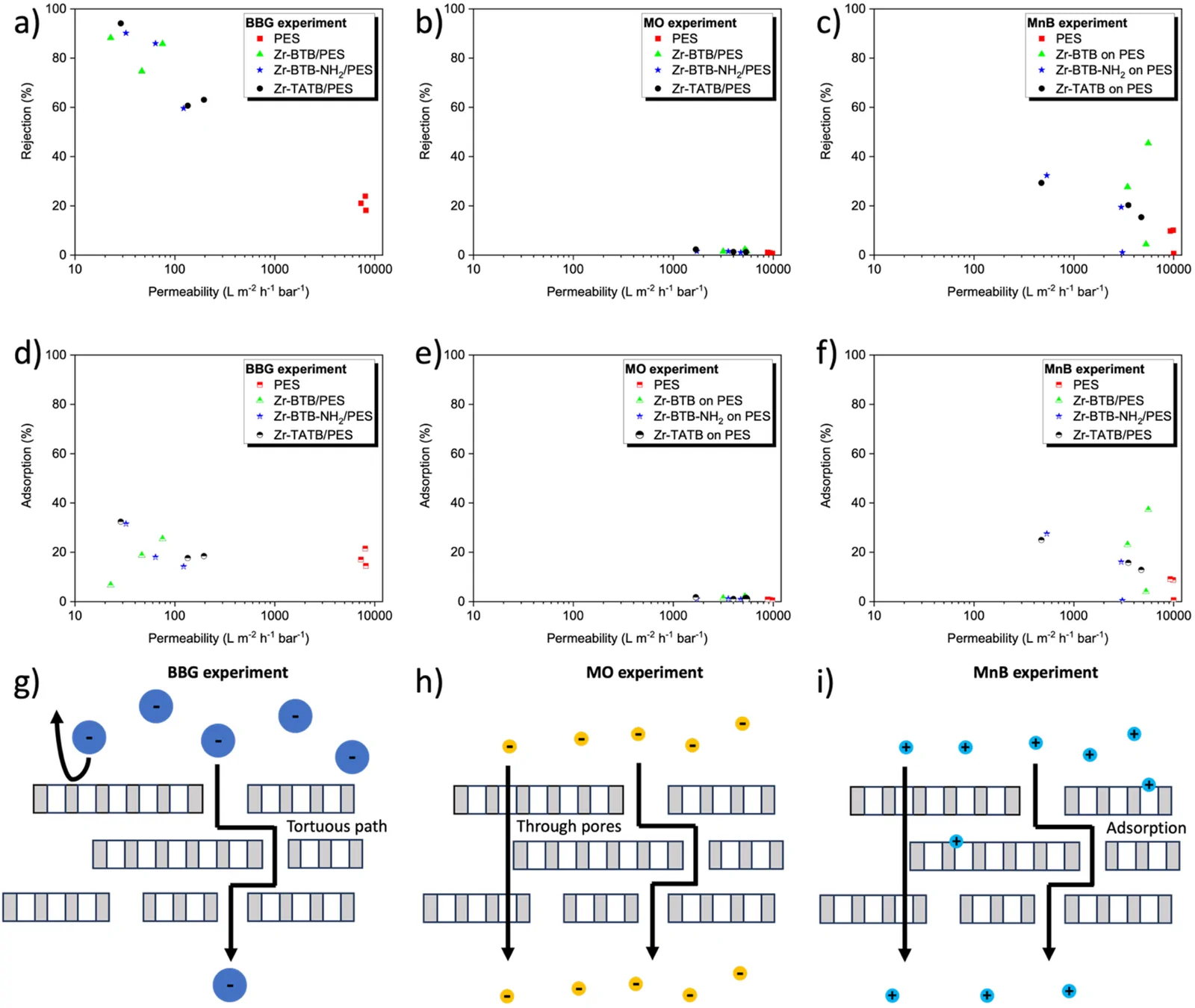
Rejection % and water permeance of the PES support, Zr–BTB/PES, Zr–BTB–NH_2_/PES and Zr–TATB/PES membranes in (a) BBG, (b) MO and (c) MnB experiments. Adsorption % and water permeance of the PES support, Zr–BTB/PES, Zr–BTB–NH_2_/PES and Zr–TATB/PES membranes in (d) BBG, (e) MO and (f) MnB experiments. Schematic illustrations showing how different dyes passed through the selective layers composed of MONs in (g) BBG, (h) MO and (i) MnB experiments.

The water purification performance of the membranes towards smaller negatively charged MO dyes was investigated. Compared to BBG, the PES supports exhibited higher water permeances of 9276 ± 515.8 L m^−2^ h^−1^ bar^−1^, but almost no rejection toward MO (0.9 ± 0.2%, Table 1). Spin-coating of the three MONs onto the PES supports did not significantly improve their rejection of MO, and the Zr–BTB/PES, Zr–BTB–NH_2_/PES and Zr–TATB/PES membranes showed rejection percentages of only 1.8 ± 0.4%, 1.4 ± 0.3%, and 1.6 ± 0.6% toward MO, respectively (Table 1).

The water purification performance of the membranes toward small positively charged MnB dyes was also studied. The PES supports exhibited permeances of 9705 ± 393.8 L m^−2^ h^−1^ bar^−1^ toward aqueous MnB solutions (Table 1), which close to their permeances toward aqueous MO solutions because the two dyes are similar in size. The removal of MnB dyes in aqueous solutions by the PES supports was found to be predominantly due to adsorption. The PES support exhibited rejection percentages of 6.9 ± 5.3% towards MnB, while the adsorption percentages of MnB for PES supports were 6.2 ± 4.8% (Table 1).

Spin-coating of the three MONs onto the PES supports decreased their water permeance but increased the adsorption of MnB onto the membranes and their rejection of MnB (Fig. 4c and f). In three repeated experiments, the Zr–BTB/PES, Zr–BTB–NH_2_/PES and Zr–TATB/PES membranes exhibited rejection percentages of 25.8 ± 20.4%, 17.7 ± 15.6%, and 21.3 ± 7.1% toward MnB dyes, respectively, and the adsorption percentages of MnB on the membranes were 21.3 ± 16.6%, 14.8 ± 13.8%, and 18 ± 6.2%, respectively (Table 1). This indicated that the removal of MnB dyes in aqueous solutions by the composite membranes predominantly came from adsorption (Fig. 4i) and the MONs deposited on the PES supports did not significantly improve their size exclusion capability towards MnB.

### 2.4 Discussion

These results indicate that both size exclusion and adsorption can affect the water purification performance of the membranes (Fig. 4g–i). For BBG, the trigonal dye (1.72 × 2.22 × 0.56 nm) is substantially larger than the crystallographic pore size of 0.54 nm.^15^ The dye must therefore traverse a highly tortuous path around the anisotropic nanosheet layers (Fig. 4g), resulting in high rejection and low permeance. For the smaller MO (1.47 × 0.43 × 0.25 nm), its linear geometry allows permeation through both the crystallographic pores and interparticle voids (Fig. 4h), explaining the near-zero rejection and higher permeance. MnB (1.46 × 0.50 × 0.18 nm) is similar in size and shape to MO so can also pass through the pores and voids, however it shows a higher adsorption to both the nanosheets and PES substrate than MO.

Zeta potential measurements of the nanosheets were undertaken to explore the effect of surface charge on performance (Table S1). Interestingly, whilst Zr–TATB showed strong negative values (−39.1 ± 9.7), Zr–BTB and Zr–BTB–NH_2_ showed slightly positive values (5.4 ± 3.3 and −3.91 ± 9.7 respectively). We note that all three nanosheets form stable dispersions in water, however in the 1 mol dm^−3^ KCl electrolyte used to run these tests, Zr–BTB and Zr–BTB–NH_2_ were observed to precipitate out during testing which may have affected these results. These differences in surface charge don't translate into significant differences in adsorption behavior between the nanosheets which show similar trends in behavior across all three systems indicating electrostatic interactions are not a dominant mechanism. The relatively high adsorption observed for both negatively charged BBG and positively charged MnB are therefore attributed to the relatively large, planar aromatic surfaces which cause them to undergo hydrophobic and π-stacking interactions. In contrast the permanently ionized sulfonate groups and lack of extended aromatic groups in negatively charged MO mean they don't adsorb strongly to the nanosheets or PES membrane.

Despite their different chemical functionalities of the ligands, all three MONs show very similar rejection, adsorption and permeance behavior across all three dyes. This is attributed to the isoreticular structure of all three MONs resulting in a consistent pore aperture and surface chemistry, which is only subtly affected by the addition of an amine or triazine group to the BTB-core. Differences caused by these functionalities may also be masked by the relatively high standard deviations associated all measurements shown in Table 1 and Fig. 4. A wide range of factors may have contributed to this, including minor differences in the underlying commercial support, the thickness, packing and uniformity of the MON layers, presence of non-selective membrane defects. Further optimisation of MON-loading and spin-coating parameters could reduce variability within the system and allow differences to be distinguished and more contrasting ligands with bulkier, charged or targeted chemical functionalities may allow greater differentiation.

Overall, this study expands the range of Zr–BTB based membranes investigated for use in water-purification applications. The use of a highly porous PES supports meant that all three membranes showed higher permeances (48.2–119.6 L m^−2^ h^−1^ bar^−1^ for BBG) than were achieved in comparable literature reports using Zr–BTB nanosheets doctor bladed onto PAN membranes (1–1–22.7 L m^−2^ h^−1^ bar^−1^).^18,32^ Although other MON based membranes showed higher selectivity's for dye separations, of the five systems reported (discussed in the introduction and summarised in SI Table S3) only Zn–TCP(Fe)–PEI/Nylon^19^ MONs showed higher permeances. Combined with the high stability of Zr–BTB MONs this makes these systems potentially attractive to develop for use in real-world applications.

## 3. Conclusions

In summary, this work confirms that the high stability, well defined pore sizes and tunable chemistry of Zr–tricarboxylate based MONs can be effectively harnessed for use in dye separation. We successfully used modulated synthesis to produce an isoreticular series of high-aspect ratio and ultrathin nanosheets in high yield and processed them into composite membranes by spin-coating onto porous PES supports. This enabled us to achieve high permeabilities (up to 119.6 L m^−2^ h^−1^ bar^−1^) compared to other Zr–MON based membranes in the literature. Notably, despite the variation in ligand structure, filtration performance remained consistent across the series, with 72.7–83% rejection of large anionic dyes but almost no rejection of smaller dyes. This establishes that the separation mechanism is robust and primarily governed by the crystallographic pore size of the framework rather than specific ligand interactions. These findings therefore validate Zr–tricarboxylate based MONs as a reliable and structurally tunable platform for high-performance water purification.

## 4. Experimental section

### 4.1. General considerations

All chemicals and solvents were obtained from commercial suppliers and used without any purification, apart from the H_3_BTB–NH_2_ and H_3_TATB ligands were synthesised using the procedure reported below. Details of all equipment used and material characterisations can be found in the SI.

### 4.2. Preparation of Zr–BTB, Zr–BTB–NH_2_ and Zr–TATB MONs

The three MONs were synthesised by adapting the method reported by Zhao and co-workers.^24^ ZrCl_4_ (0.129 mmol) and tritopic carboxylic acid ligands (H_3_BTB, H_3_BTB–NH_2_ or H_3_TATB, 0.068 mmol) were dissolved in DMF (15 mL), H_2_O (2 mL), and HCOOH (2 mL) in a glass reaction vial, which was placed in a reaction oven and heated at 120 °C for 24 hours. The resulting suspensions of the three MONs were centrifuged and washed three times with fresh DMF then ethanol to give a yield between 22–39%. The washed MONs were stored in fresh ethanol as homogeneous suspensions with a concentration of 2.5 mg mL^−1^ until needed.

### 4.3 Preparation of water purification membranes

The membranes were prepared by depositing two 500 µL portions of ethanol suspension of Zr–BTB, ZrBTB–NH_2_ or Zr–TATB MONs (2.5 mg mL^−1^) onto PES supports using a spin-coating method. The spin-coating of all membranes was conducted at 2500 rpm for 640 s using an Ossila spin coater. The PES supports used in this work were purchased from Sterlitech Corporation (diameter: 47 mm, pore size: 200 nm). All membranes were dried at room temperature before characterisations and water purification experiments.

### 4.4 Water purification experiments

The membranes were fixed in a Sterlitech HP4750 stirred cell for the water purification experiments (Fig. S16). The cell was pressurised using N_2_ gas at 1 bar, and the feed solution was stirred at 500 rpm during the experiments. For each experiment, 0.25 L of aqueous BBG, MO or MnB solution (10 mg L^−1^) was used as the feed which was filtered by the membrane and the experiment stopped when approximately 0.2 L of permeate was collected. The concentrations of the feed, permeate and retentate were measured by an UV-vis spectrophotometer, and the water purification experiment was repeated three times for each membrane. The water permeance, rejection and adsorption of the membranes were calculated using equations reported in the literature^19^ and the results are summarised in Table 1.

The water permeance, *F*, of each membrane is calculated by eqn (1).1
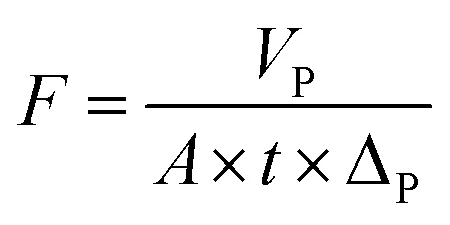
where, *V*_P_ is the volume of the permeate (unit: L), *A* is the effective area of the membrane (unit: m^2^), *t* is the time of collecting approximately 0.2 L of permeate solution (unit: h), and *Δ*_P_ is the operating pressure (unit: bar). Thus, the unit of *F* is L m^−2^ h^−1^ bar^−1^. In this work, *A* and *Δ*_P_ are 0.00146 m^2^ and 1 bar, respectively.

The rejection %, *R*, of each membrane is calculated by eqn (2).2
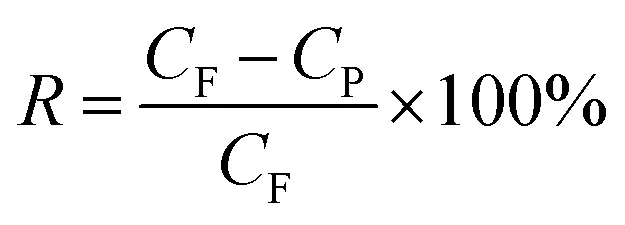
where, *C*_F_ and *C*_P_ are the concentrations of the feed and the permeate solutions, respectively.

The adsorption %, Ads of each membrane is calculated by eqn (3).3
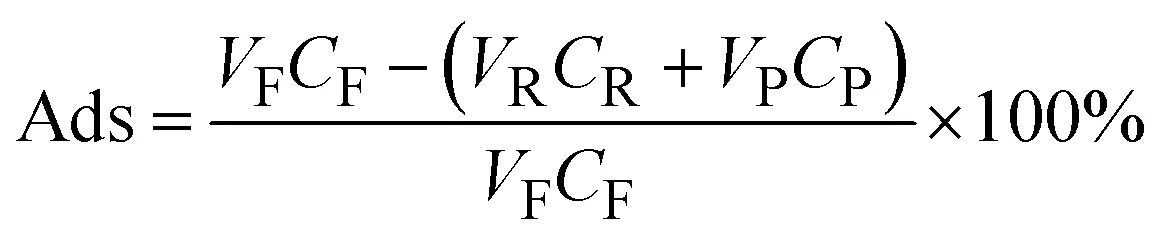
where, *V*_F_, *V*_P_ and *V*_R_ are the volumes of the feed, permeate, and retentate solutions, respectively. *C*_F_, *C*_P_ and *C*_R_ are the concentrations of the feed, permeate and retentate solutions, respectively. In this work, *V*_F_, *V*_P_ and *V*_R_ are approximately 0.25 L, 0.2 L and 0.05 L, respectively.

## Conflicts of interest

The authors declare that they have no known competing financial interests or personal relationships that could have appeared to influence the work reported in this paper.

## Supplementary Material

NA-OLF-D6NA00215C-s001

## Data Availability

Supplementary information (SI): synthesis of the MONs and membranes and providing additional characterisation and performance data and literature comparisons. The equipment and software, ligand synthesis, additional characterisations of MONs and membranes, models of organic dyes, *etc*. can be found. See DOI: https://doi.org/10.1039/d6na00215c.
