# Supplementary material for: An isoreticular series of Zr–tricarboxylate based metal–organic nanosheets for water purification applications
Source: Nanoscale Adv. 2026 Sep 9. Online ahead of print. doi: 10.1039/d6na00215c (PMC13629479; doi:10.1039/d6na00215c)
Supplement: Supplementary file 1 [file NA-OLF-D6NA00215C-s001.pdf]

Supplementary Information (SI) for Nanoscale Advances.
This journal is © The Royal Society of Chemistry 2026

Supplementary Information

An isoreticular series of Zr-tricarboxylate based metal-organic nanosheets for water purification applications

Jiangtian Tan,*[a] Bethany J. Lingard,[b] Ram R. R. Prasad,[b] and Jonathan A. Foster*[b]

[a]*Centre for Molecular Systems and Organic Devices (CMSOD), State Key Laboratory of Flexible Electronics (LoFE) & Institute of Advanced Materials (IAM), Nanjing University of Posts & Telecommunications, 9 Wenyuan Road, Nanjing 210023, China.*

[b]*School of Mathematical and Physical Sciences, The University of Sheffield, Sheffield S3 7HF, U.K*

*Joint corresponding authors.

E-mail address: jona.foster@sheffield.ac.uk (J. A. Foster)

Table of contents

S1. Equipment and Software 2

S1.1. Atomic force microscopy 2

S1.2. Dynamic light scattering 2

S1.3. Powder X-ray diffraction 2

S1.4. Scanning electron microscopy 2

S1.5. Ultraviolet-visible spectroscopy 3

S1.6 Zeta potential analysis 3

S2. Ligand Synthesis 3

S2.1. Synthesis of 2'-amino-5'-(4-carboxyphenyl)-[1,1':3',1''-terphenyl]-4,4''-dicarboxylic acid (H_3BTB-NH_2) 3

S2.2. Synthesis of 4,4',4''-s-triazine-2,4,6-triyl-tribenzoic acid (H_3TATB) 4

S3. MONs and Membranes 5

S4. Water Purification Experiments 13

S5. Models of Organic Dyes 18

References 22

S1. Equipment and Software

S1.1. Atomic force microscopy

Atomic force microscopy (AFM) images were recorded using a Bruker Multimode 5 Atomic Force Microscope, operating in soft-tapping mode in air under standard ambient temperature and pressure, fitted with Bruker OTESPA-R3 silicon cantilevers. AFM samples were prepared by drop-casting 10 µL of diluted ethanol suspensions of metal-organic nanosheets (MONs) onto the centre of freshly cleaved mica sheets heated to 120 °C on a hot plate. These mica sheets were stuck to stainless steel, magnetic Agar scanning probe microscopy specimen discs. All AFM images reported in this work were processed using Gwyddion software.

S1.2. Dynamic light scattering

Dynamic light scattering (DLS) data for the MONs were collected using a Malvern ZETA SIZER Nano series instrument, using a He-Ne laser at 633 nm, operating in backscatter mode (173°). Polystyrene disposable cuvettes were used for the DLS experiments, and the temperature for the DLS experiments was set to 25 °C.

S1.3. Powder X-ray diffraction

Powder X-ray diffraction (PXRD) data of the MONs and the membranes were collected using a Bruker-AXS D8 diffractometer using Cu K_α (λ=1.5418 Å) radiation and a LynxEye position sensitive detector in Bragg Brentano parafocussing geometry using a flat silicon plate.

S1.4. Scanning electron microscopy

Scanning electron microscopy (SEM) samples were prepared by loading the MONs and membranes onto carbon sticky tabs placed on aluminium SEM sample stubs, coated with gold using an Edwards S150B gold sputter coater and loaded into a TESCAN VEGA3 LMU SEM. All SEM images and EDS element layered images reported in this work were collected using the secondary electron detector.

S1.5. Ultraviolet-visible spectroscopy

Ultraviolet-visible (UV-Vis) absorption spectra were collected using a Cary 60 or a Cary 50 Probe UV-Vis instrument, using a 1 cm internal length quartz cuvette and the Cary WinUV, Scan software.

S1.6 Zeta potential analysis

Samples were MON suspended in 1 mol dm-3 KCl solution, with the sample concentration equivalent to 1 mg mL-1. Samples were sonicated for 2 hours at 37 kHz, 100% intensity. Zeta potential data was collected using the same Malvern Zetasizer Nano Series particle size analyser as DLS in zeta potential mode. Disposable polycarbonate capillary electrophoresis cells were used, according to the Smoluchowski method. Samples were equilibrated at 298 K for 20 s before analysis.

S2. Ligand Synthesis

S2.1. Synthesis of 2′-amino-5′-(4-carboxyphenyl)-[1,1′:3′,1′′-terphenyl]-4,4′′-dicarboxylic acid ($H_3BTB-NH_2$)

The synthesis of $H_3BTB-NH_2$ was carried out following a modified approach based on reported procedures [1]. A mixture of 2,4,6-tribromoaniline (2.5 g, 7.5 mmol), p-ethoxycarbonylphenyl boronic acid (6 g, 30 mmol), sodium carbonate (4.3 g, 40 mmol), and tetrakis(triphenylphosphine)palladium(0) (0.6 g, 0.5 mmol) were added into a 500 mL N_2-purged round-bottom flask. Then, degassed toluene/MeOH/H_2O (160 mL, v/v/v = 2:1:1) was added to the reaction mixture using a cannula, which was heated to reflux under an N_2 atmosphere. After completion of the reaction, the solvent was removed under vacuum, and the residue was extracted with dichloromethane (3 × 80 mL). The extracts were washed with brine (80 mL) and dried over anhydrous $MgSO_4$. The crude product was purified by flash column chromatography to afford 1,3,5-tri(p-methoxycarbonyl-phenyl)aniline, which was then hydrolysed with 6 M NaOH (30 mL) in a mixture of THF and MeOH (100 mL, v/v = 1:1) to yield $H_3BTB-NH_2$ as a yellowish solid.

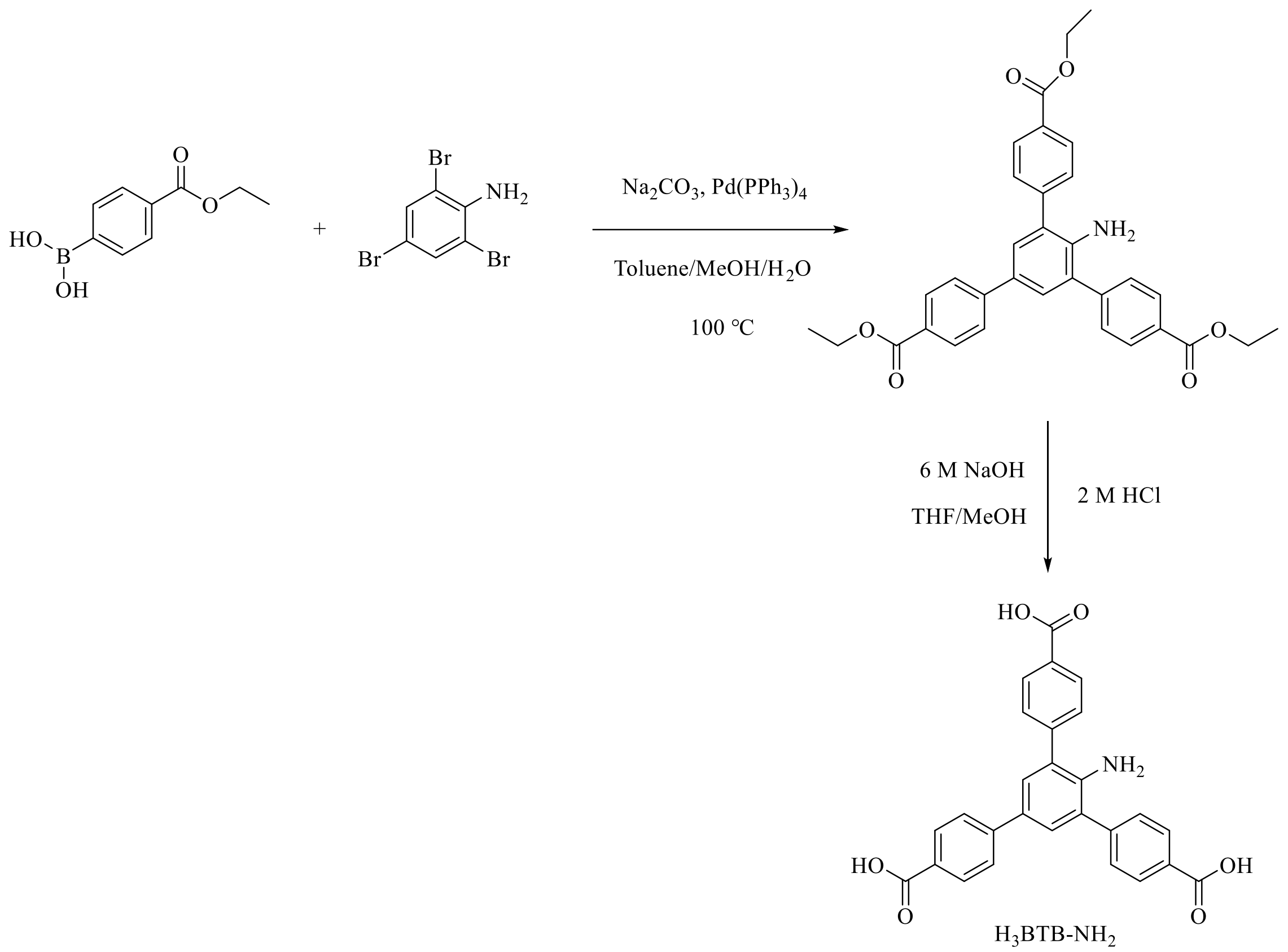

Scheme 1: Synthesis of $H_3BTB-NH_2$.

S2.2. Synthesis of 4,4',4''-s-triazine-2,4,6-triyl-tribenzoic acid (H_3TATB)

The synthesis of H_3TATB was carried out following a reported procedure [2]. **Synthesis of 2,4,6-tri-*p*-tolyl-1,3,5-triazine (TTT):** To 14.63 mL (166 mmol) of trifluoromethanesulfonic acid in a round-bottom flask, 7 g (60 mmol) of 4-methylbenzonitrile was added slowly in portions over a period of 2 h. After the mixture was stirred at room temperature overnight, the resulting orange solution was poured into ice-water, neutralized with an aqueous ammonia solution, and stirred for 2 h. The white precipitate that formed was collected by filtration, washed multiple times with a water-acetone mixture (v/v = 1:1), and dried overnight at 80 °C. Recrystallization from hot toluene afforded white needle-shaped TTT crystals. **Synthesis of H_3TATB:** 2.78 g (7.9 mmol) of TTT and 70 mL of acetic acid were added into a 250 mL round-bottom flask equipped with a magnetic stirrer. Then, 4.4 mL of H_2SO_4 was added slowly until the solution became clear. The flask was then placed in an ice-water bath, and a solution of 7.2 g (72 mmol) of $Cr(VI)O_3$ in 4.8 mL of acetic anhydride was added dropwise. After complete addition, the reaction mixture was stirred for 15 minutes. The ice-water bath was then removed, and the resulting dark brown slurry was stirred at room

temperature overnight. The mixture was poured into 300 mL of water, stirred for 2 h, and the solid was collected by filtration and washed thoroughly with copious amounts of water. The crude solid was dissolved in 200 mL of NaOH solution (2 M), and the solution was filtered to remove any unreacted starting materials. The filtrate was acidified with HCl (1 M) to yield H_3TATB as a white solid, which was washed with water several times, and dried at 80 °C overnight.

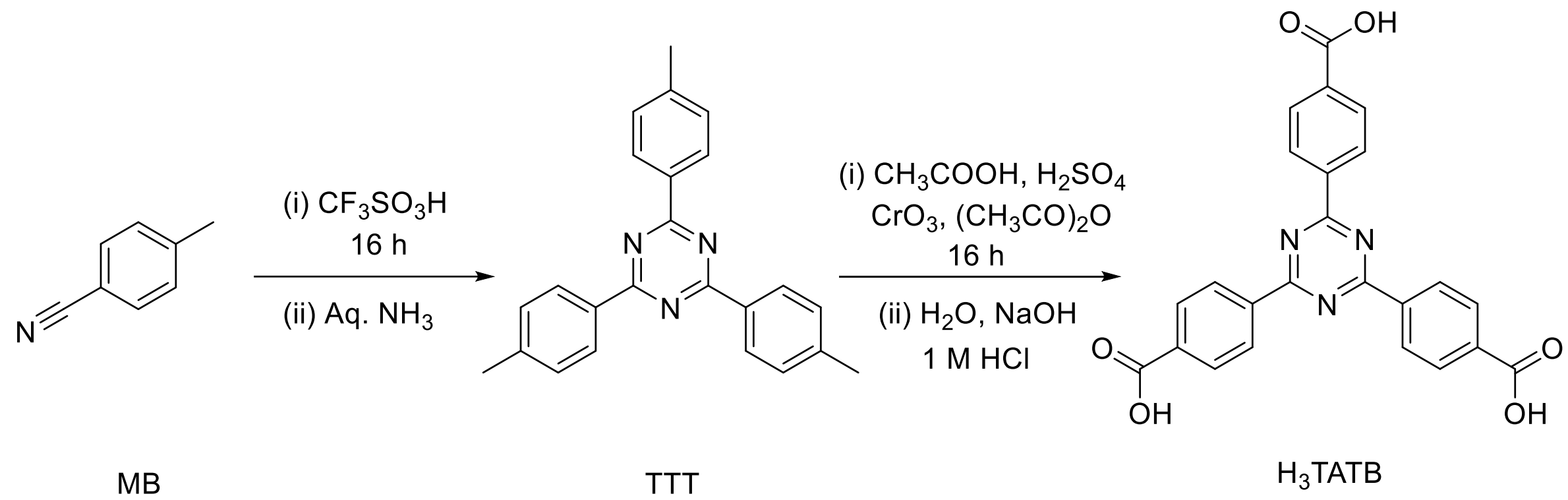

Scheme 2: Synthesis of H_3TATB.

S3. MONs and Membranes

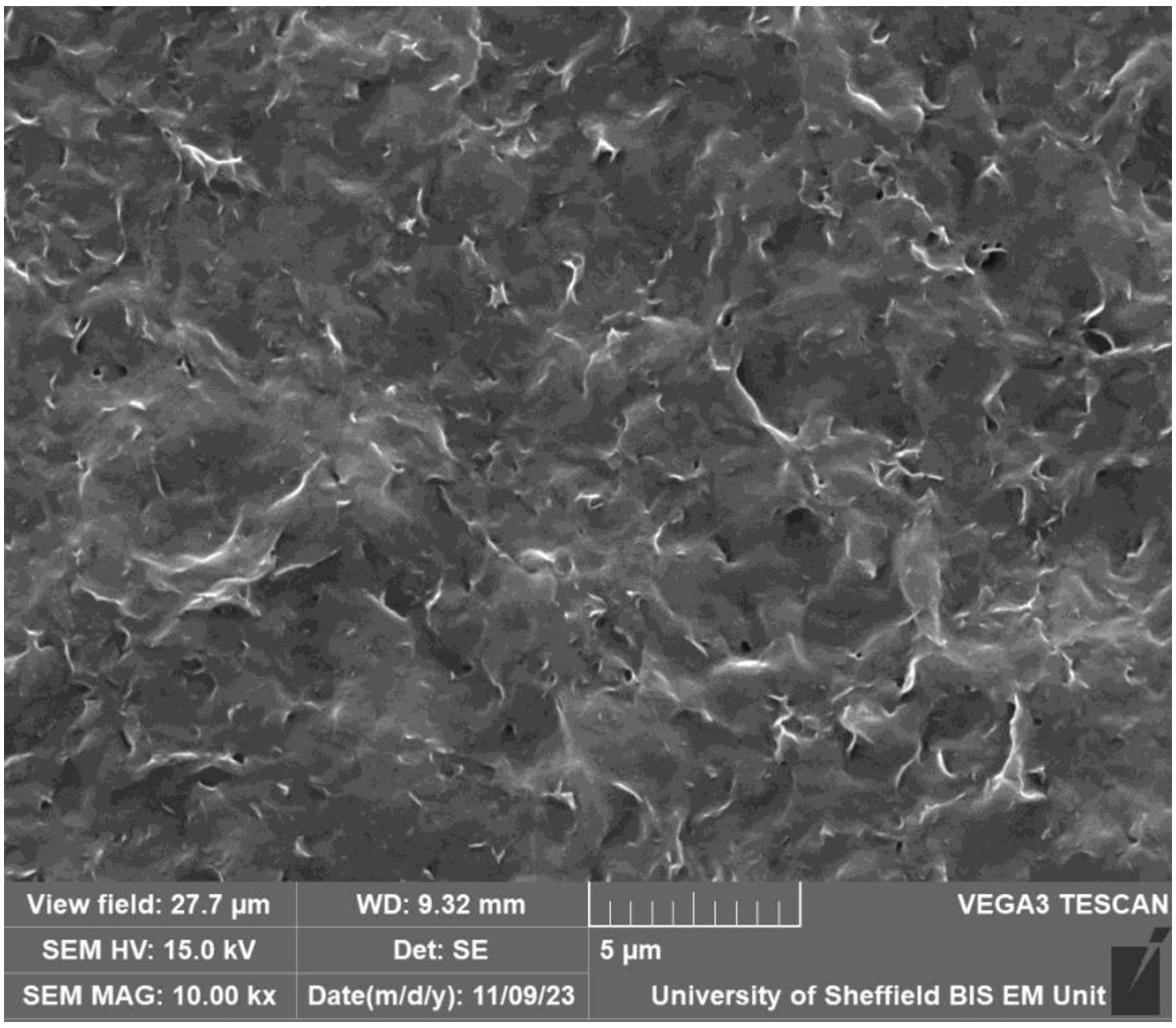

Figure S1. SEM image of Zr-BTB MONs.

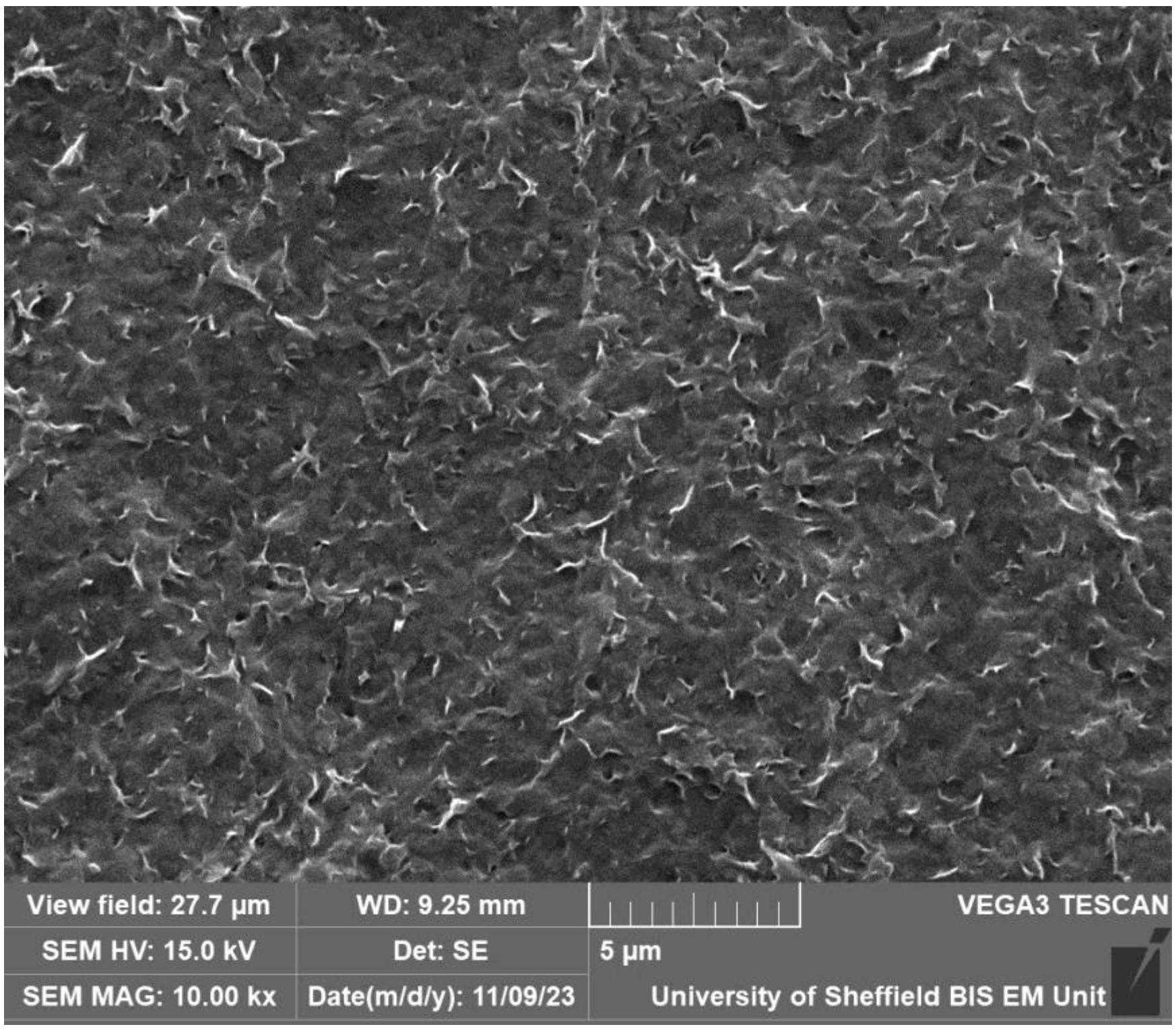

Figure S2. SEM image of Zr-BTB-NH_2 MONs.

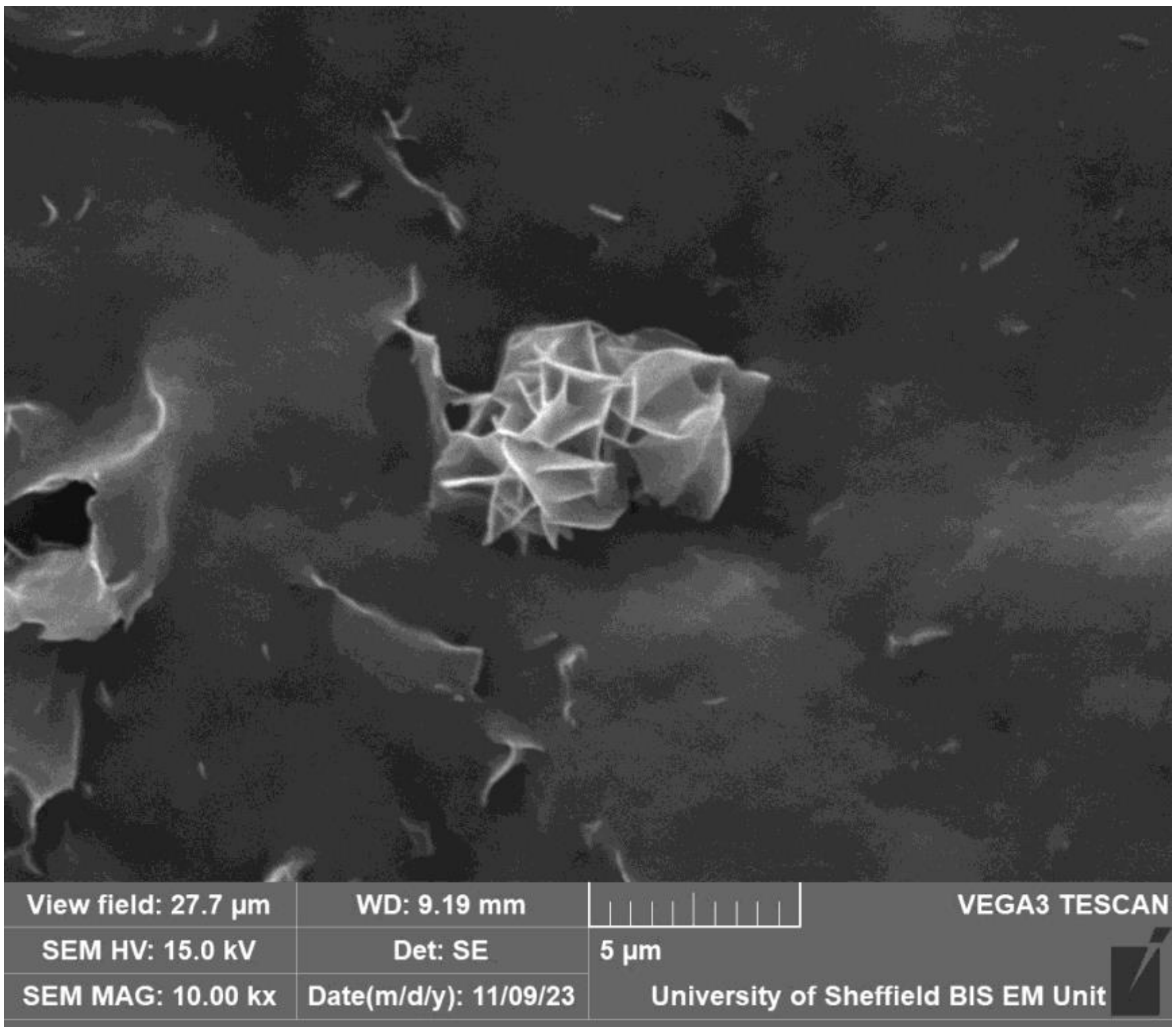

Figure S3. SEM image of Zr-TATB MONs.

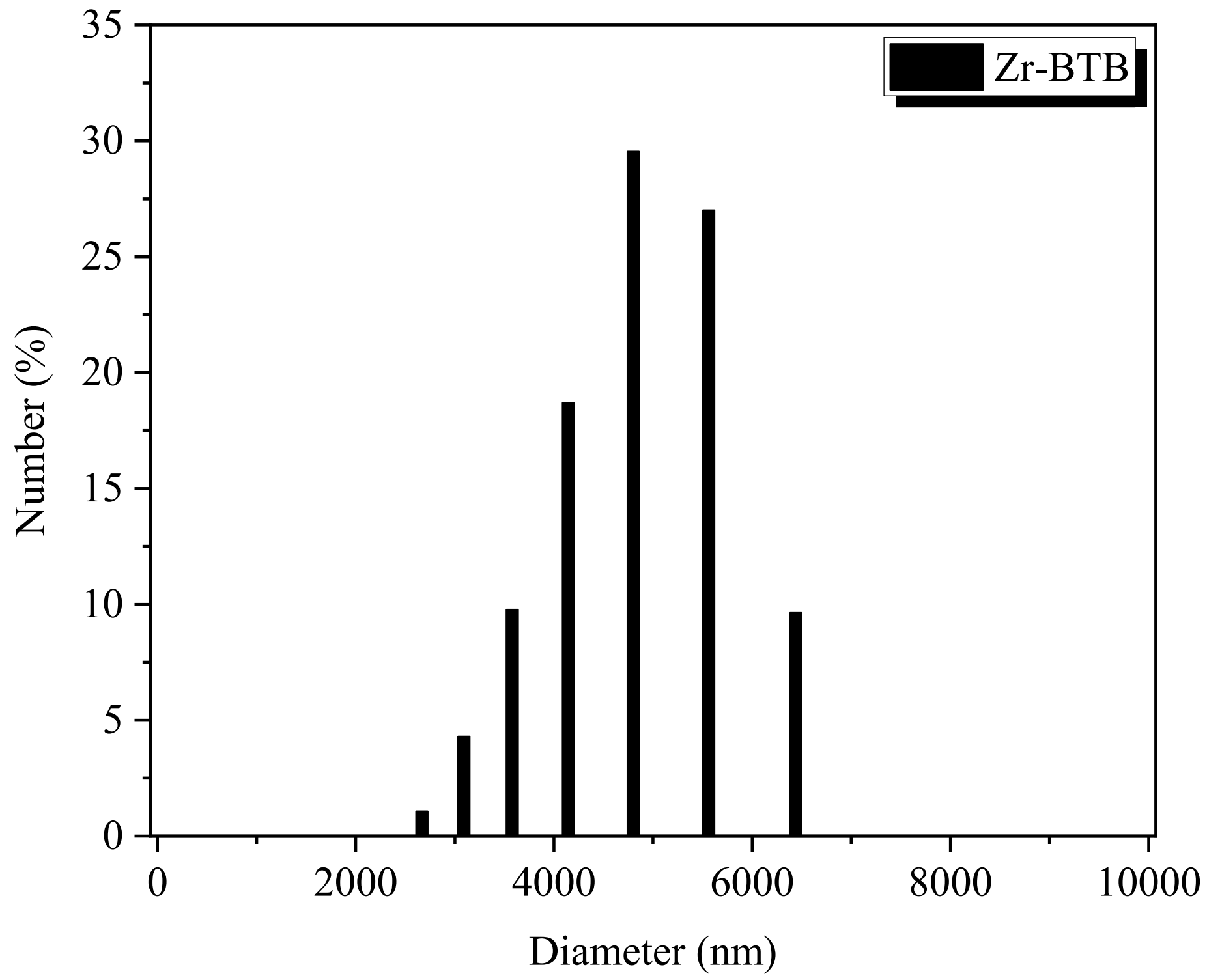

Figure S4. DLS graph showing particle size distribution of Zr-BTB MONs.

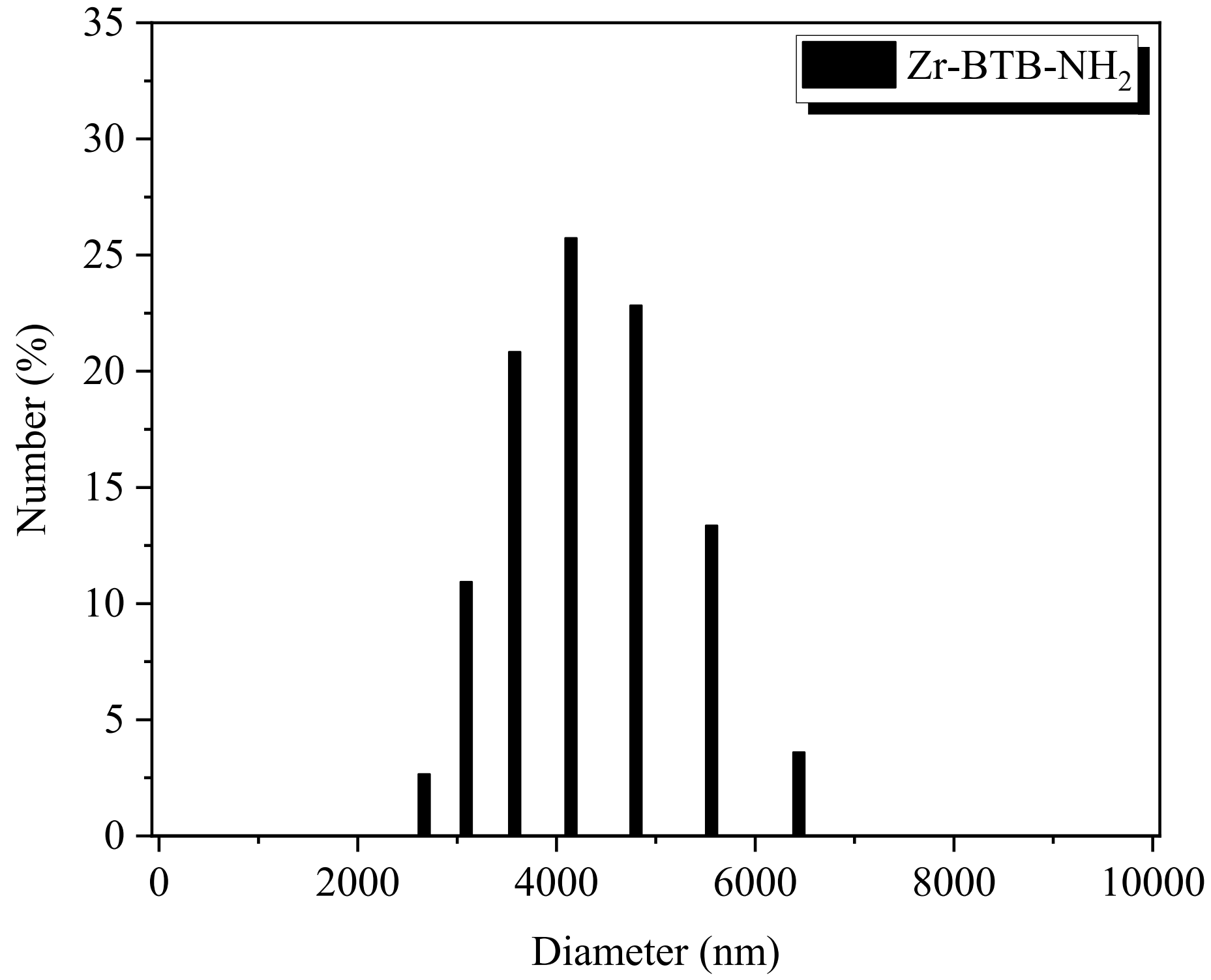

Figure S5. DLS graph showing particle size distribution of Zr-BTB-NH_2 MONs.

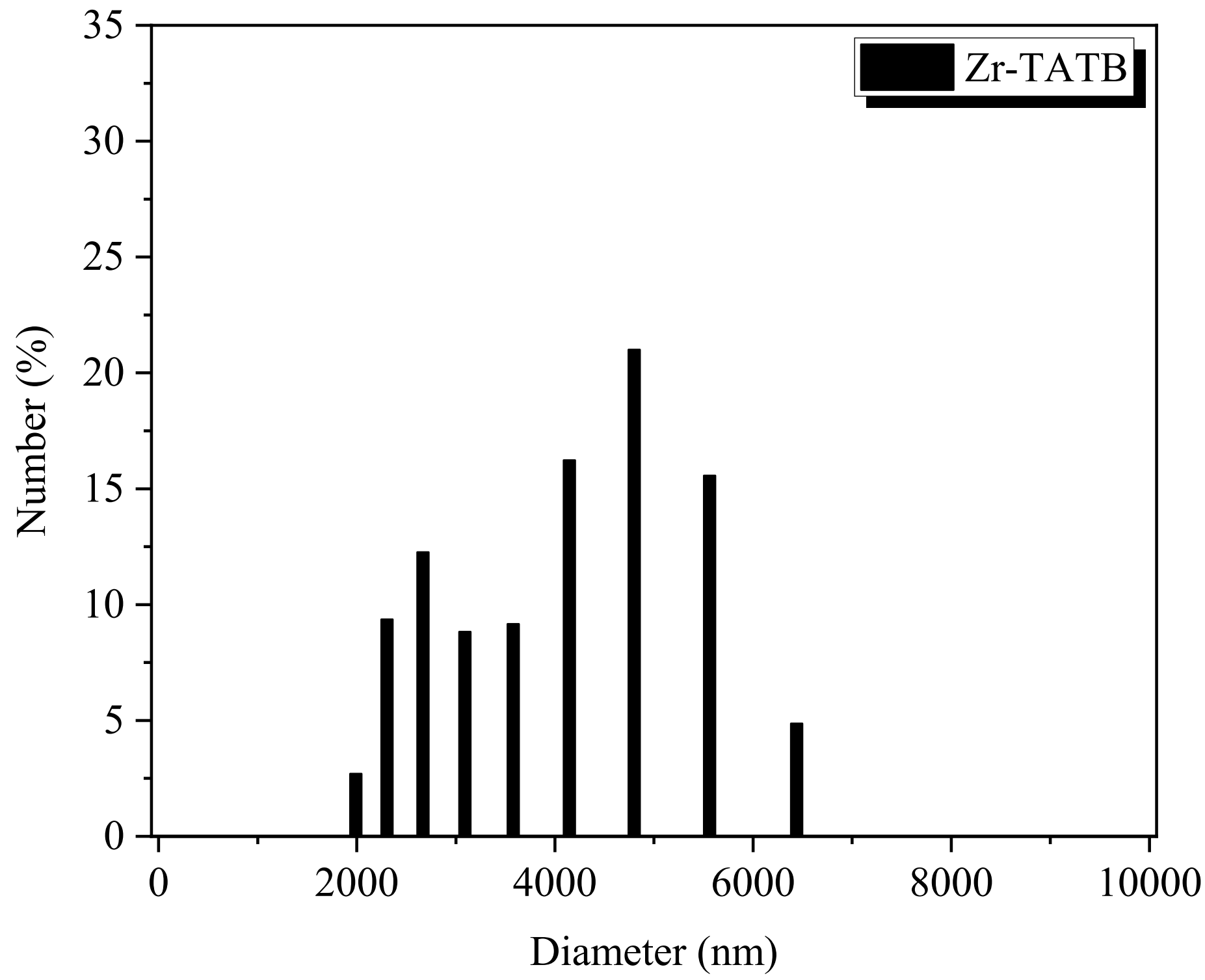

Figure S6. DLS graph showing particle size distribution of Zr-TATB MONs.

Table S1. *Zeta potentials of Zr-BTB, Zr-BTB-NH_2, and Zr-TATB MONs.*

MON	Zeta potential (mV)
Zr-BTB	7.2 ± 4.3
Zr-BTB-NH_2	5.4 ± 3.3
Zr-TATB	-39.1 ± 9.7

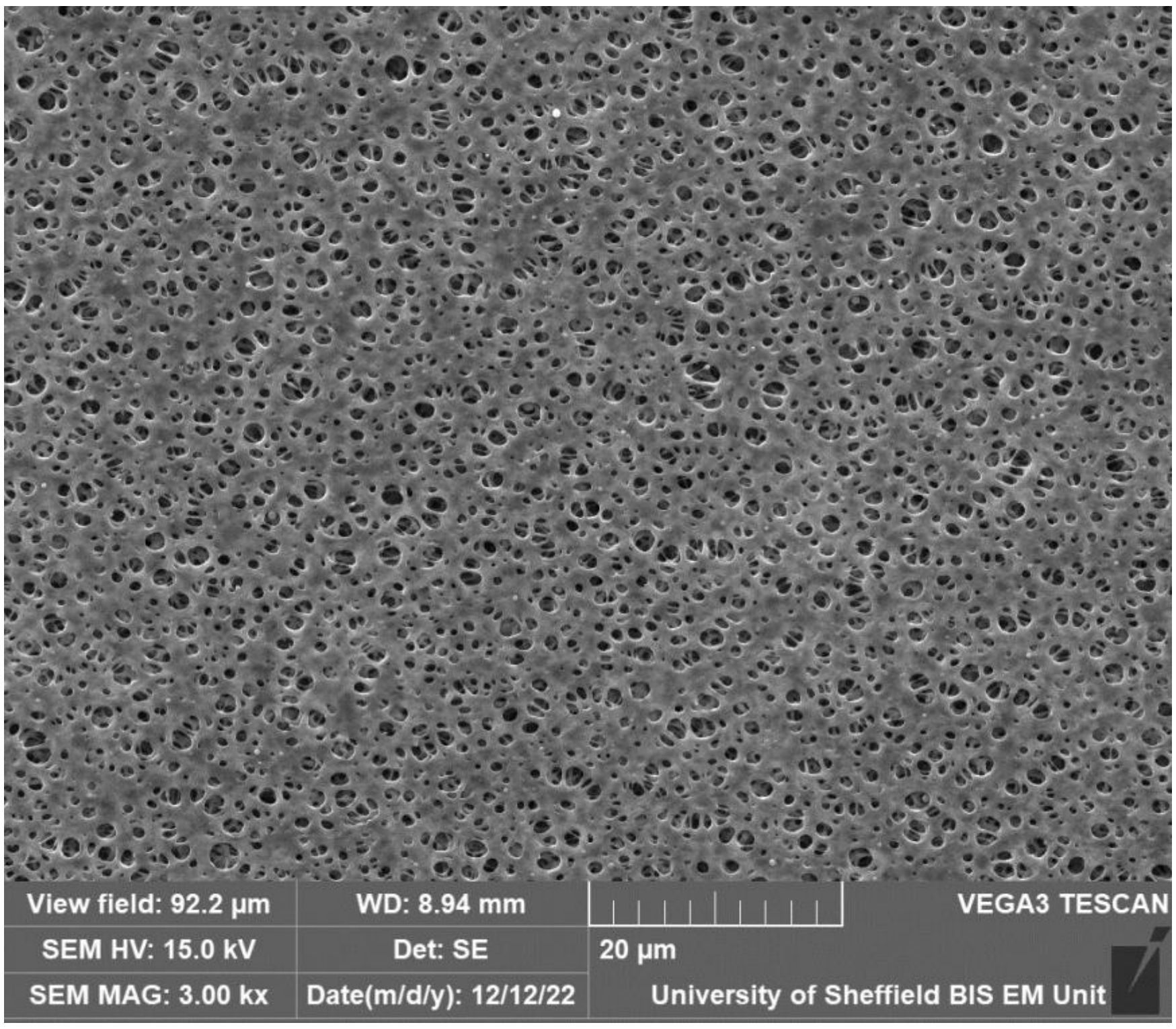

Figure S7. SEM image of PES support.

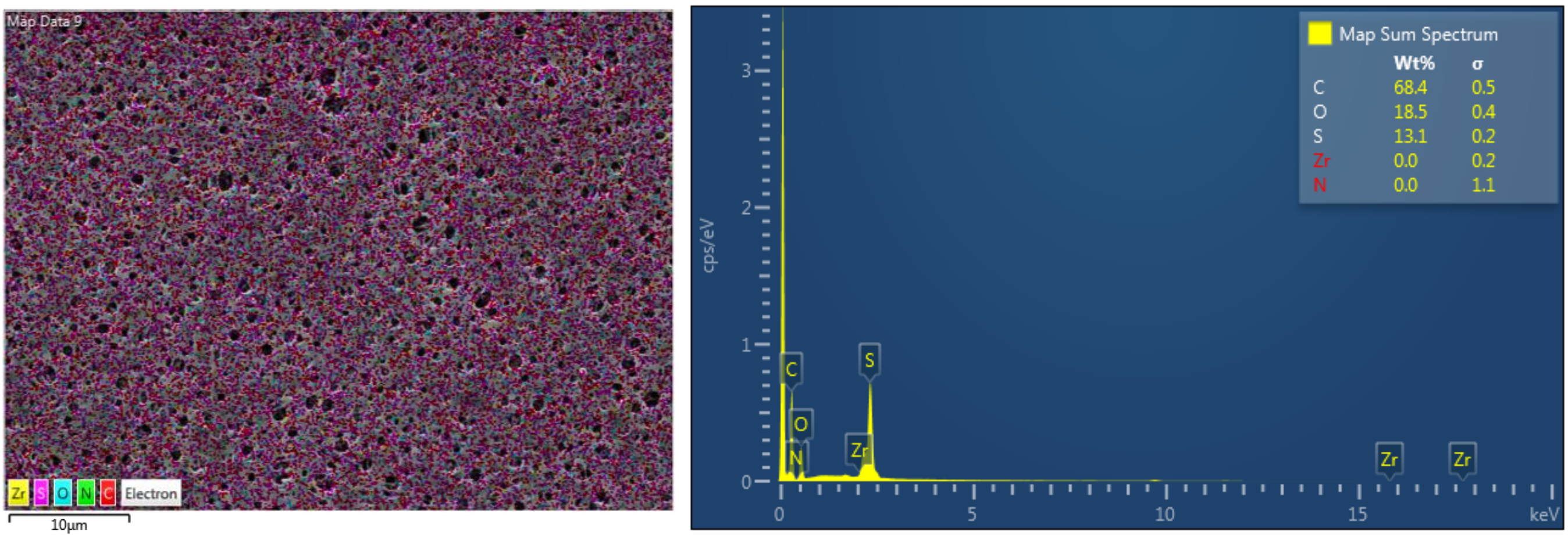

Figure S8. EDS element layered image of PES support and corresponding EDS spectrum.

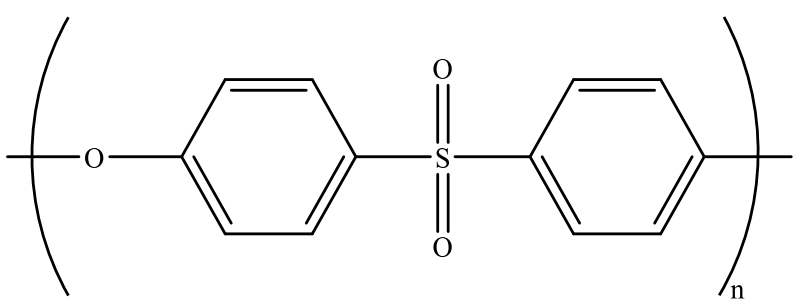

Figure S9. Chemical structure of polyethersulfone (PES).

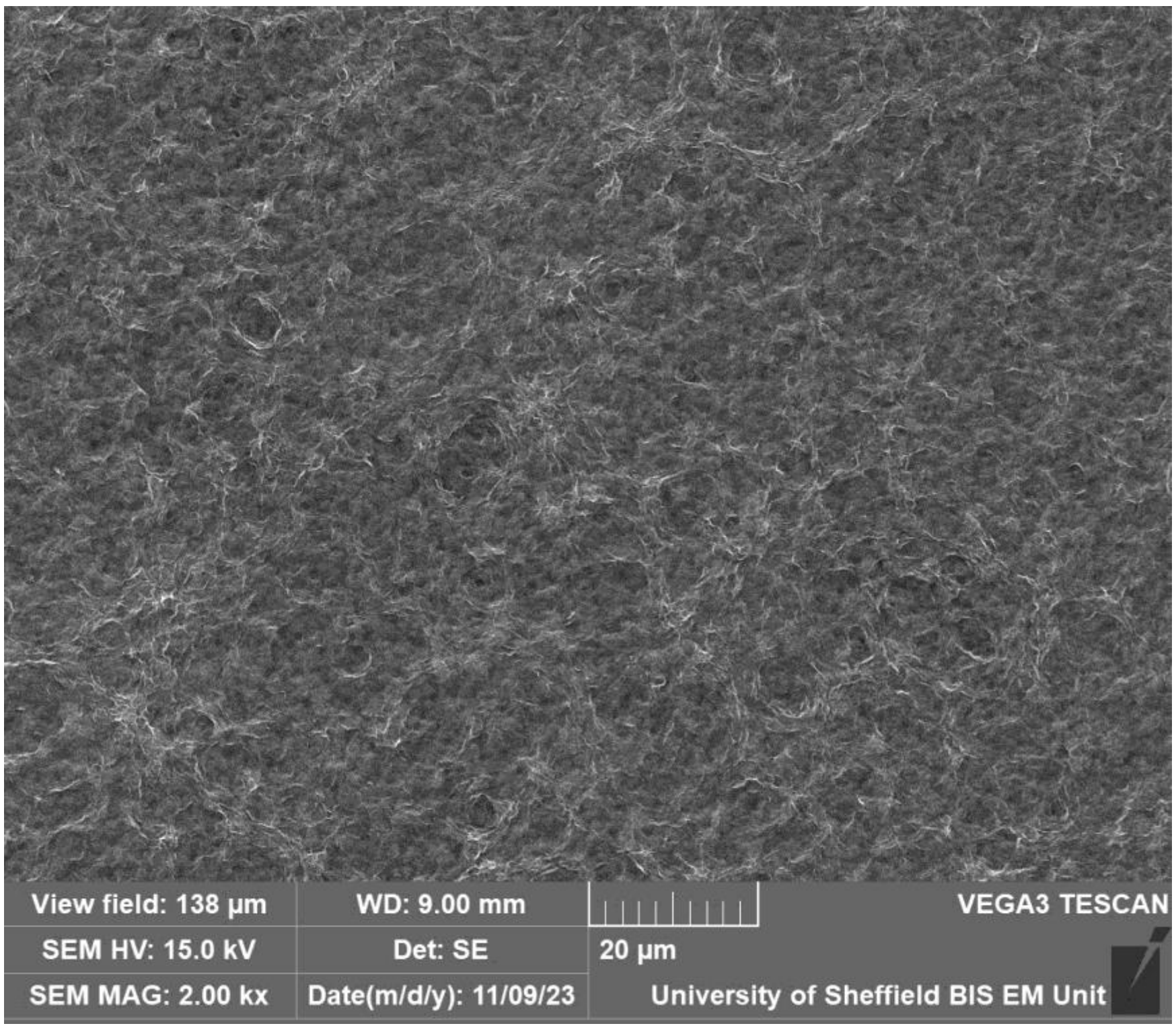

Figure S10. SEM image of Zr-BTB/PES membrane.

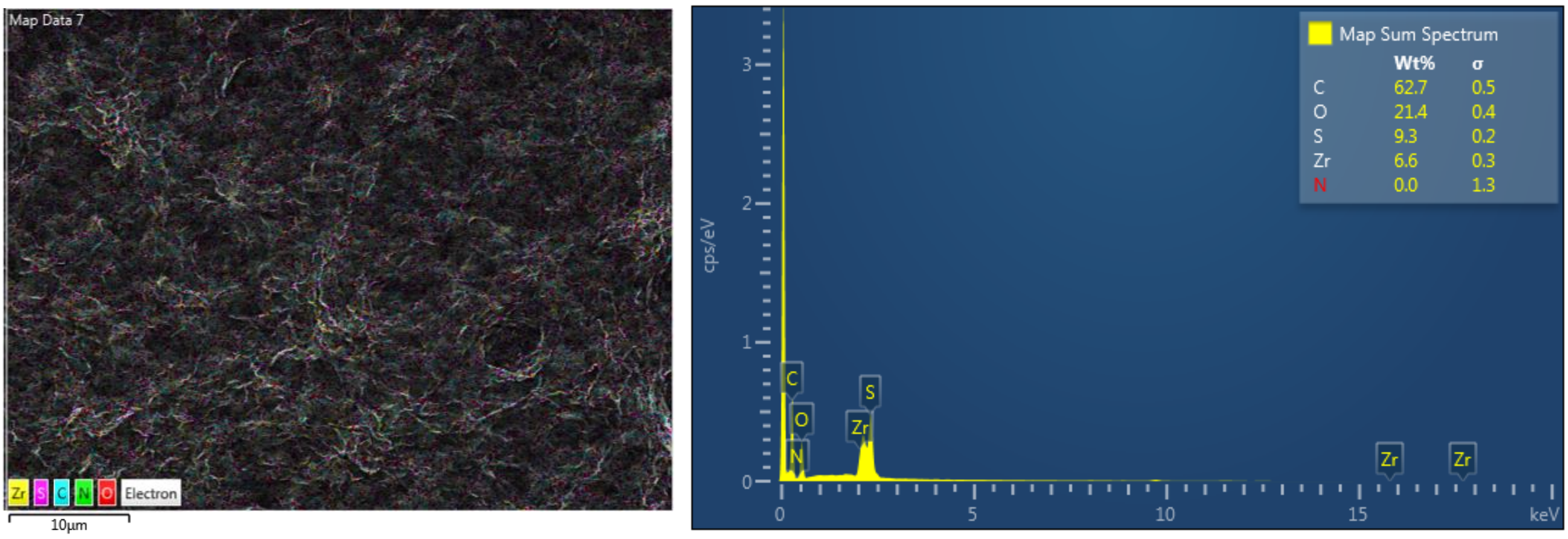

Figure S11. EDS element layered image of Zr-BTB/PES membrane and corresponding EDS spectrum.

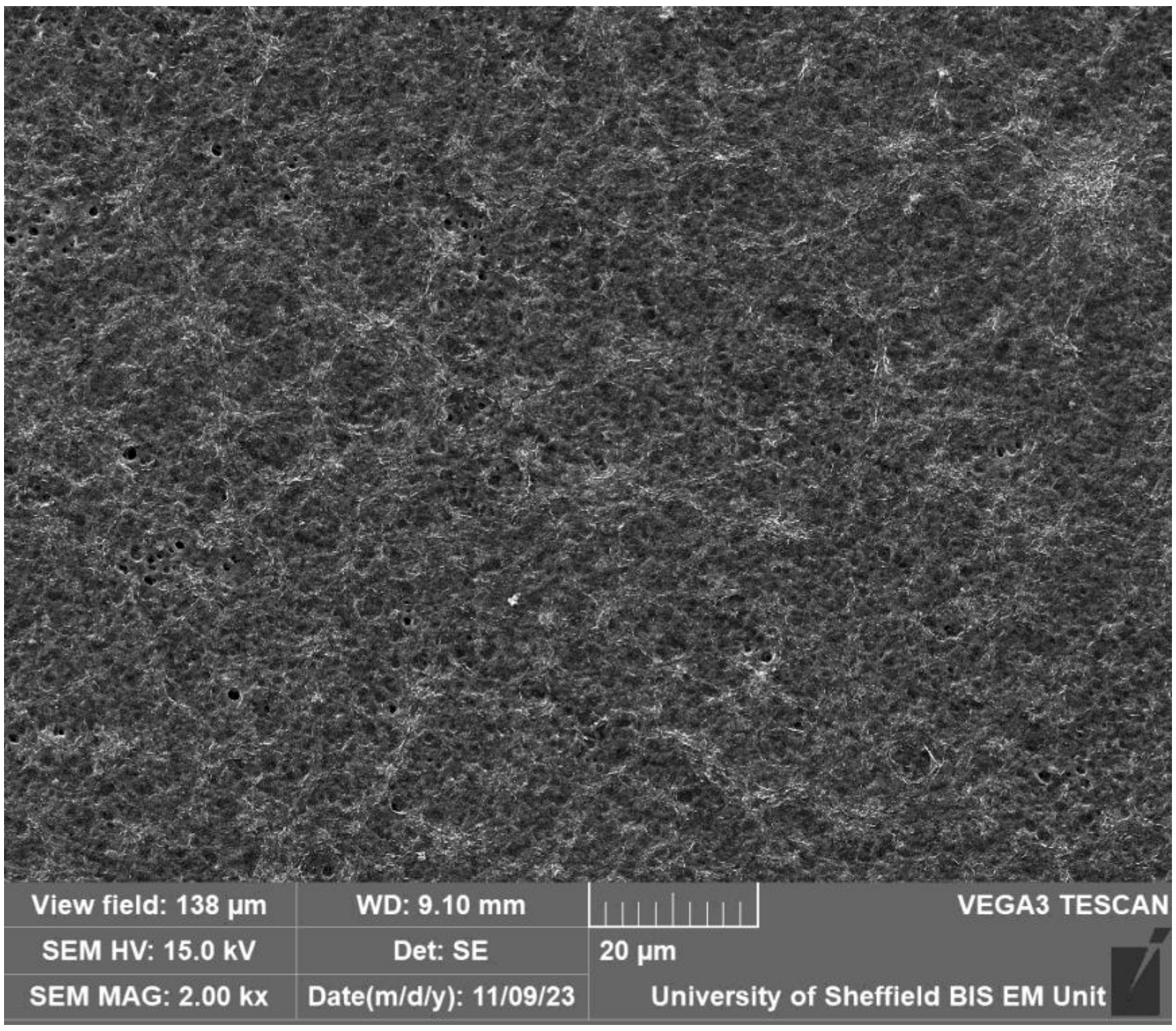

Figure S12. SEM image of Zr-BTB-NH_2/PES membrane.

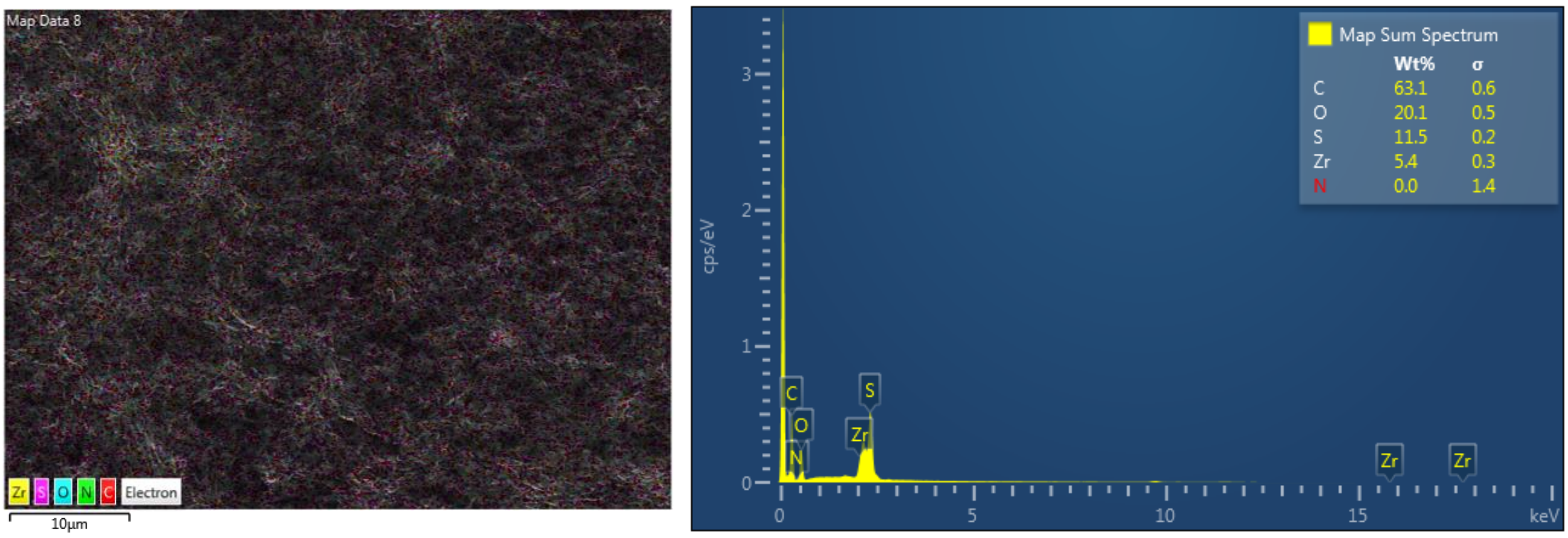

Figure S13. EDS element layered image of Zr-BTB-NH_2/PES membrane and corresponding EDS spectrum.

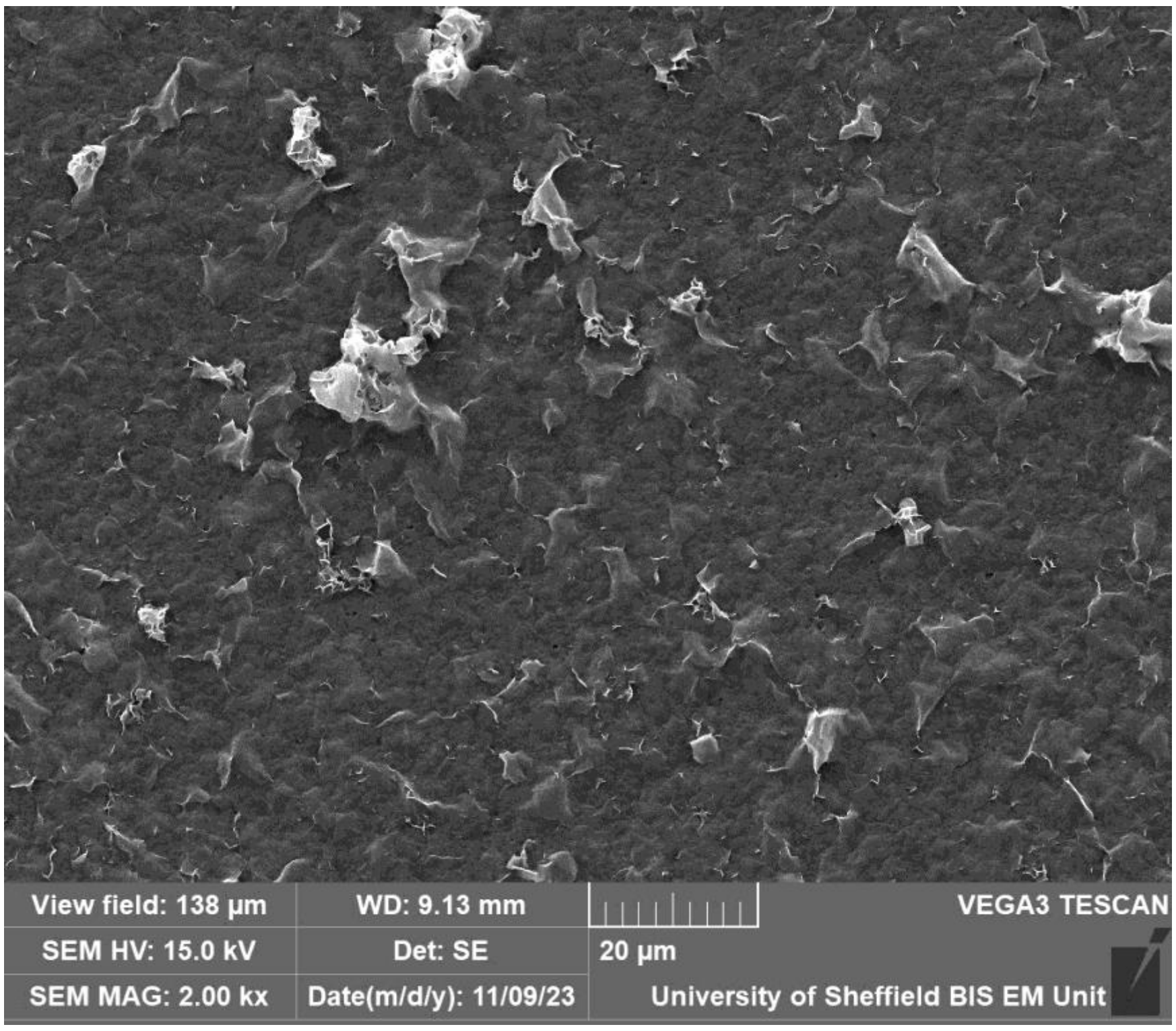

Figure S14. SEM image of Zr-TATB/PES membrane.

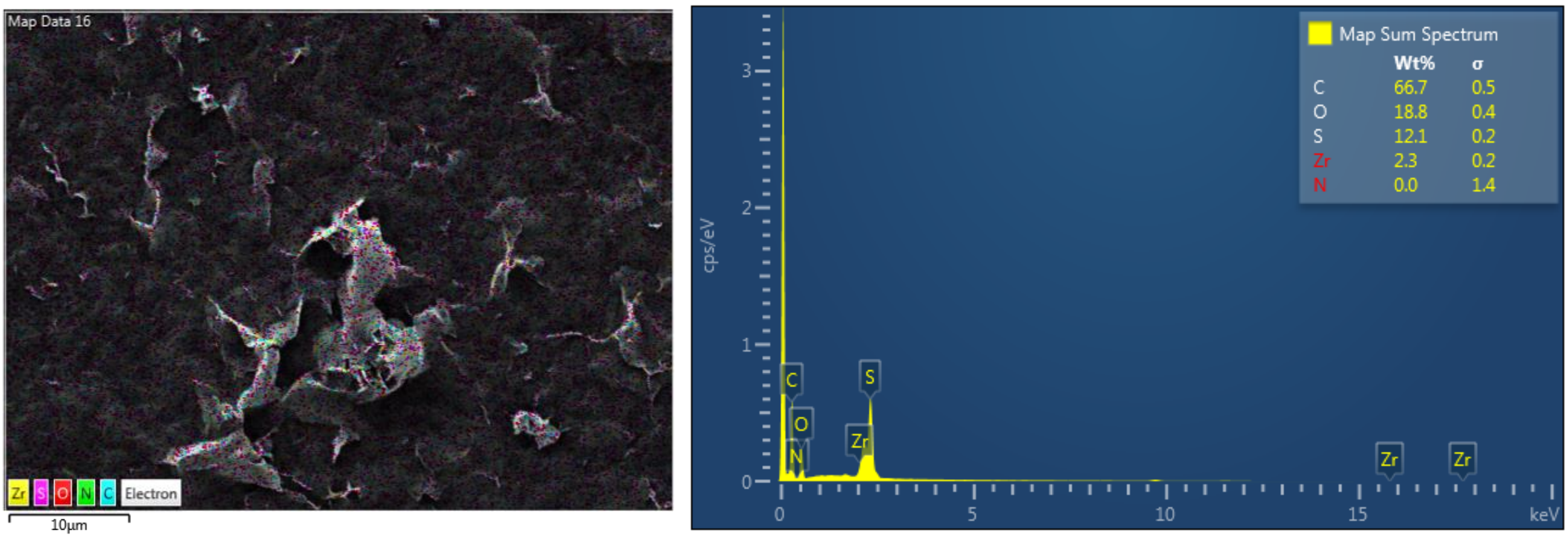

Figure S15. EDS element layered image of Zr-TATB/PES membrane and corresponding EDS spectrum.

S4. Water Purification Experiments

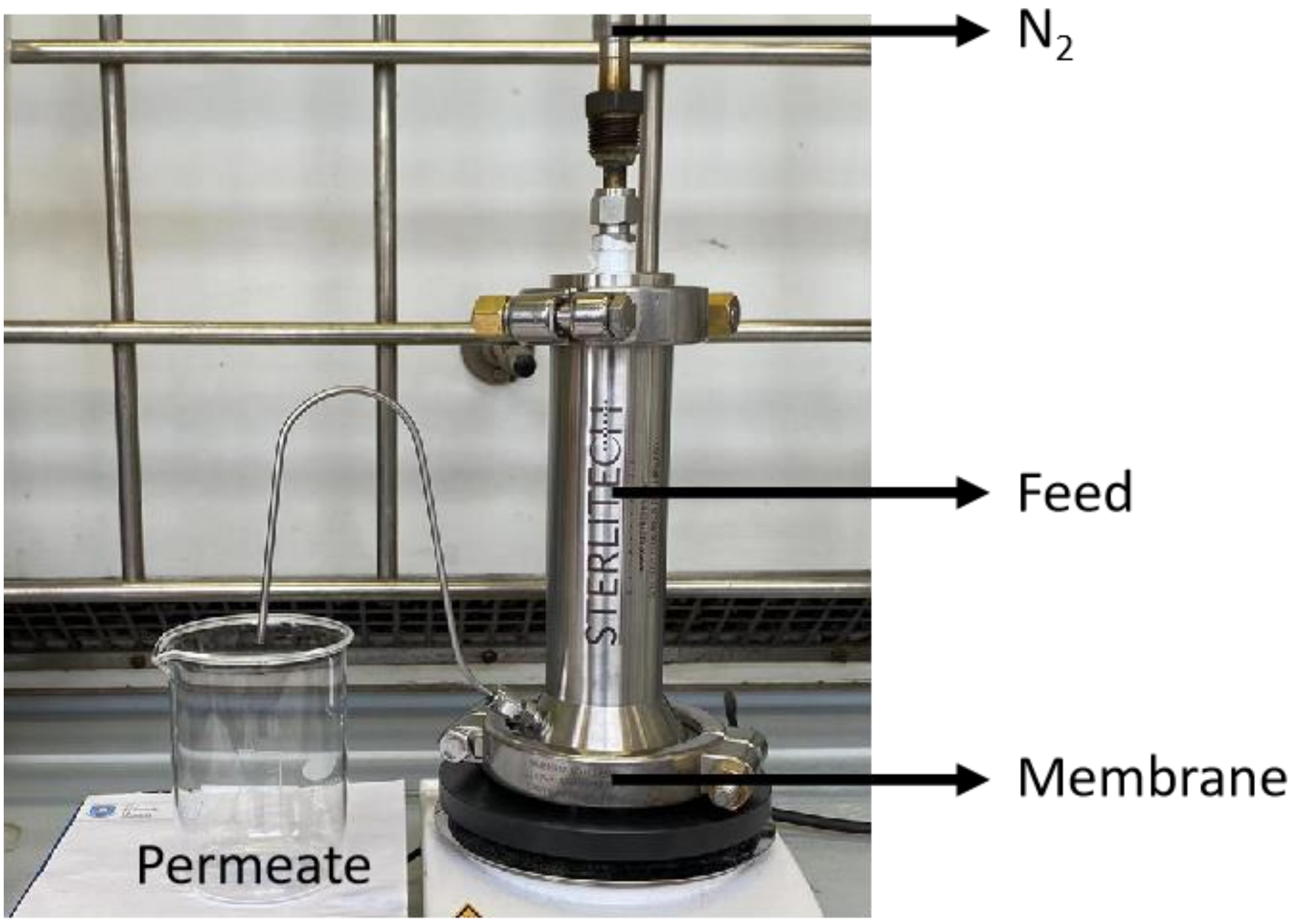

Figure S16. The Sterlitech HP4750 stirred cell for water purification experiments.

Table S2. *Structures and properties of BBG, MO and MnB dyes and water purification performance of membranes.*

Dye	Structure	Properties	Membrane	Water permeance ($L\ m^{-2}\ h^{-1}\ bar^{-1}$)	Rejection (%)	Adsorption (%)
**BBG**	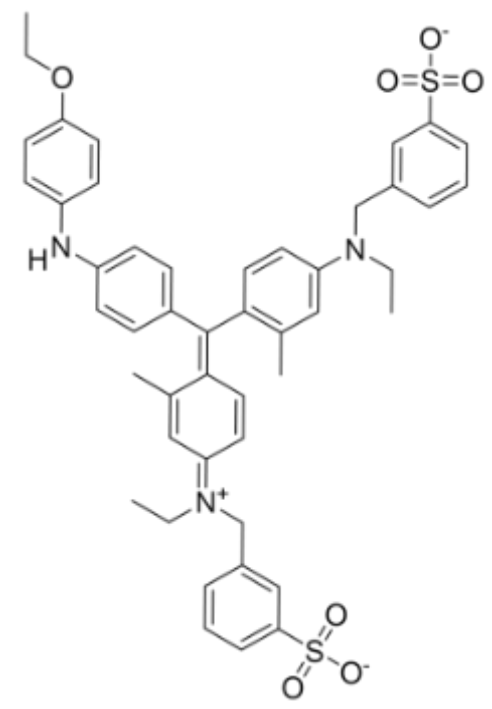	**Molecular weight (g mol^{-1})**	PES	7270	21	17
			PES	8153	18	15
			PES	8042	24	22
		831.03	Zr-BTB/PES	22.8	88	7.0
		**Dimensions of hydrated BBG* (nm)**	Zr-BTB/PES	46.7	75	19
			Zr-BTB/PES	75.2	86	26
			Zr-BTB-NH_2/PES	122.3	60	14
			Zr-BTB-NH_2/PES	64.0	86	18
		1.72 × 2.22 × 0.56	Zr-BTB-NH_2/PES	32.4	90	32
		**Formal charge**	Zr-TATB/PES	195.5	63	19
			Zr-TATB/PES	134.5	61	18
		-1	Zr-TATB/PES	28.8	94	32
**MO**	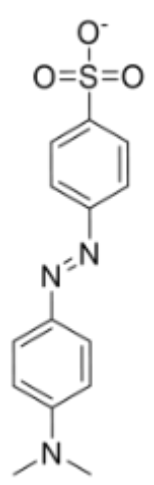	**Molecular weight (g mol^{-1})**	PES	9233	1.0	0.8
			PES	9812	0.7	0.6
			PES	8783	1.1	0.9
		304.34	Zr-BTB/PES	5435	1.6	1.4
		**Dimensions of hydrated MO* (nm)**	Zr-BTB/PES	3158	1.5	1.4
			Zr-BTB/PES	5206	2.3	2.1
			Zr-BTB-NH_2/PES	1718	1.6	1.1
			Zr-BTB-NH_2/PES	3552	1.6	1.2
		1.47 × 0.43 × 0.25	Zr-BTB-NH_2/PES	4726	1.1	0.9
		**Formal charge**	Zr-TATB/PES	1681	2.3	1.8
			Zr-TATB/PES	5347	1.3	1.1
		-1	Zr-TATB/PES	3996	1.3	1.1
**MnB**			PES	9888	10	8.8

	**Molecular weight (g mol^{-1})**	PES	9974	0.7	0.7
		PES	9253	9.9	9.1
	284.40	Zr-BTB/PES	3449	28	23
	**Dimensions of hydrated MnB* (nm)**	Zr-BTB/PES	5291	4.4	4.0
		Zr-BTB/PES	5568	45	37
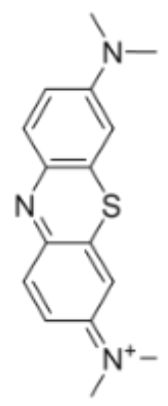		Zr-BTB-NH_2/PES	2985	20	16
		Zr-BTB-NH_2/PES	3063	1.1	0.5
	1.46 × 0.50 × 0.18	Zr-BTB-NH_2/PES	536.2	32	28
	**Formal charge**	Zr-TATB/PES	4733	15	13
		Zr-TATB/PES	3514	20	16
	+1	Zr-TATB/PES	473.1	29	25

*Dimensions of hydrated BBG, MO and MnB dyes were reported by Ang and Hong [3].

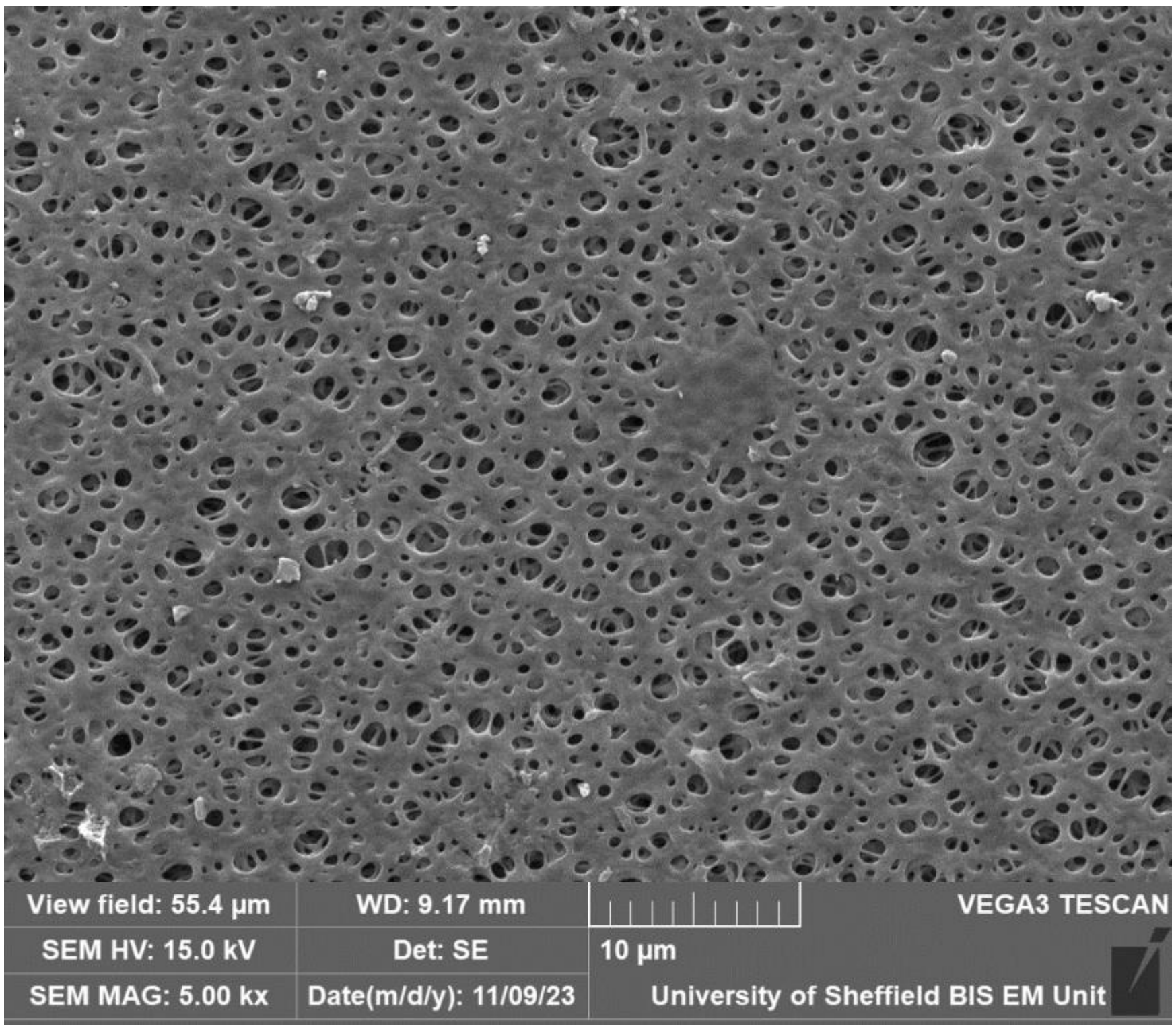

Figure S17. SEM image of PES support after BBG separation experiment.

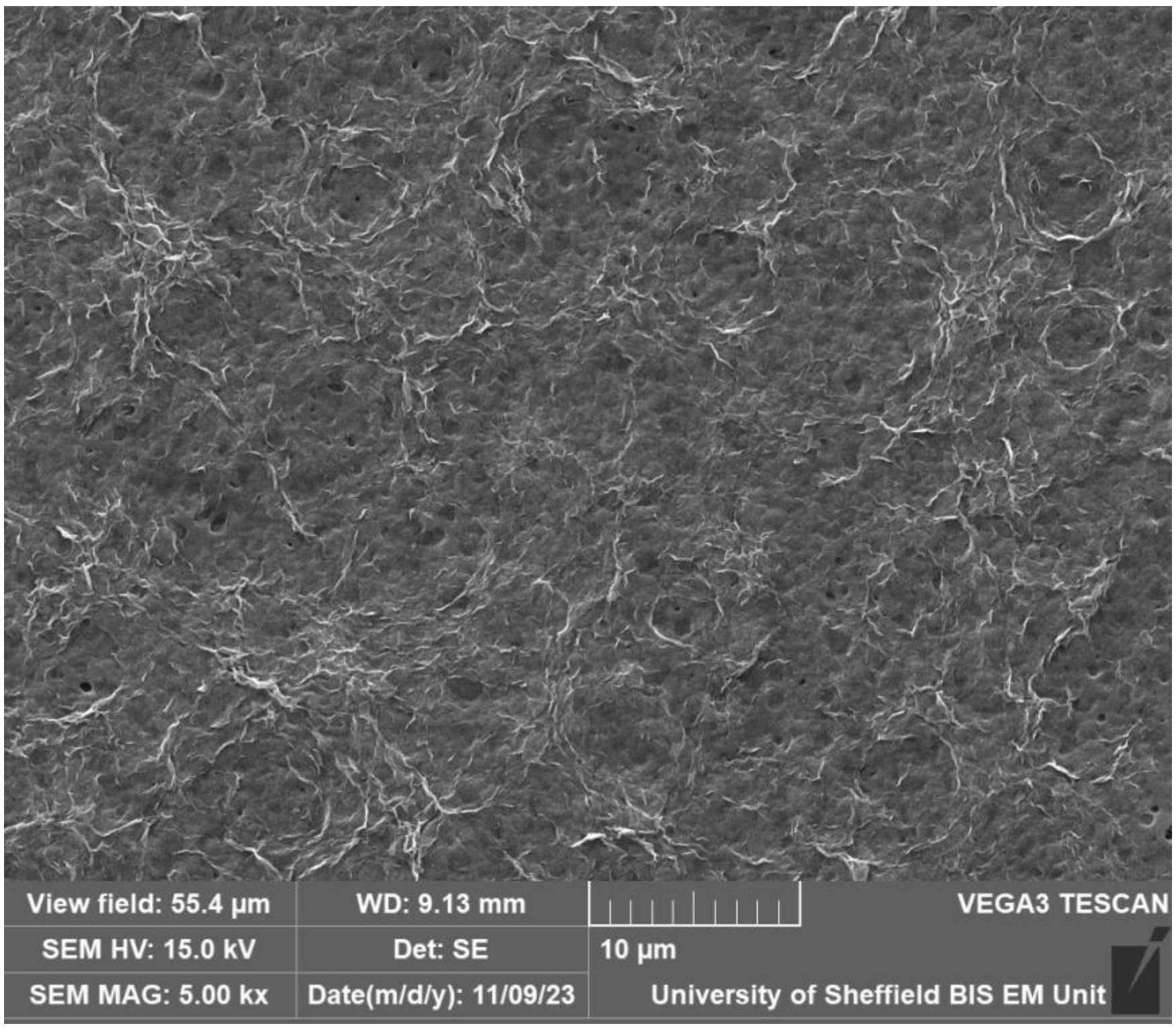

Figure S18. SEM image of Zr-BTB/PES membrane after BBG experiment.

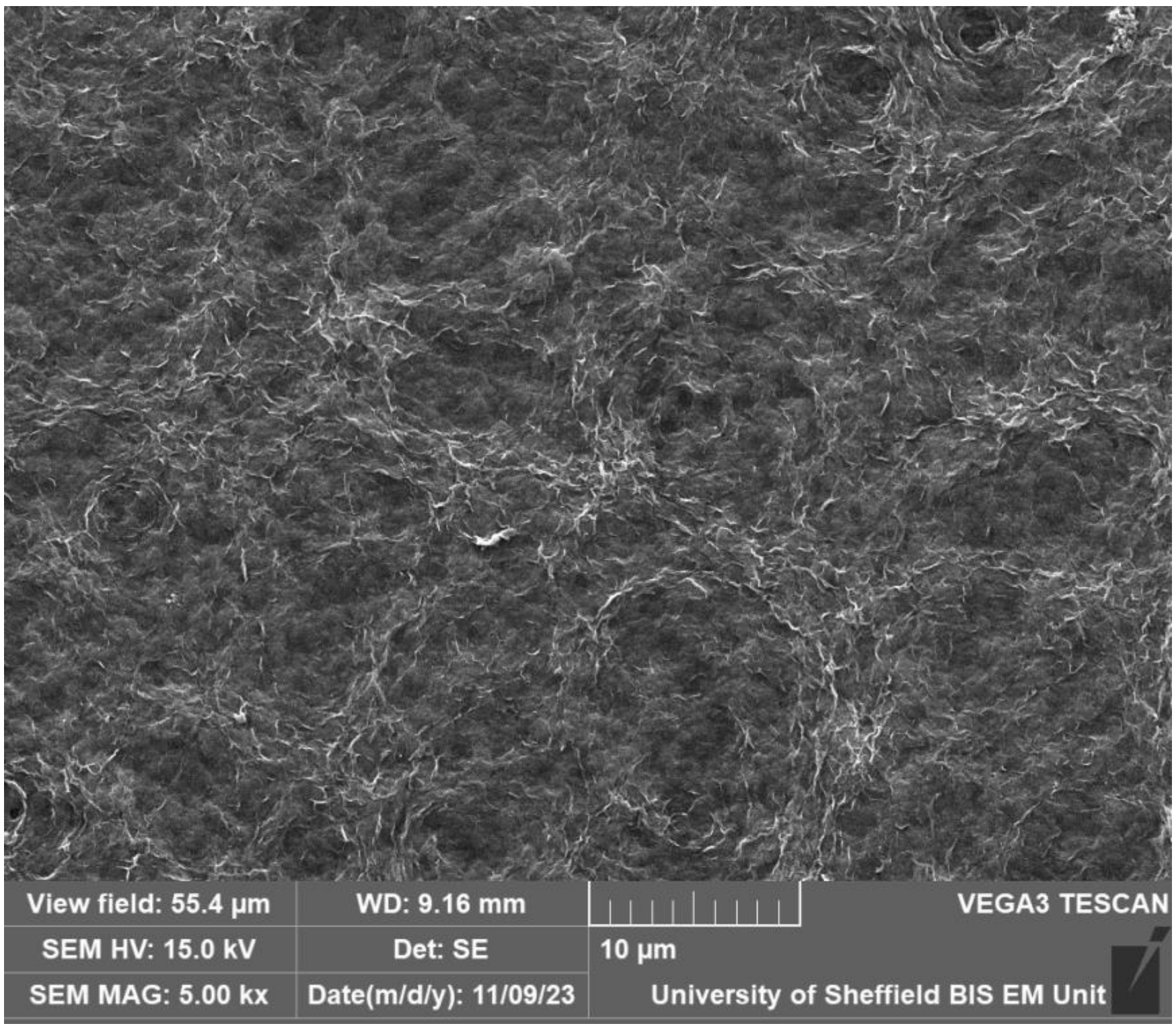

Figure S19. SEM image of Zr-BTB-NH_2/PES membrane after BBG experiment.

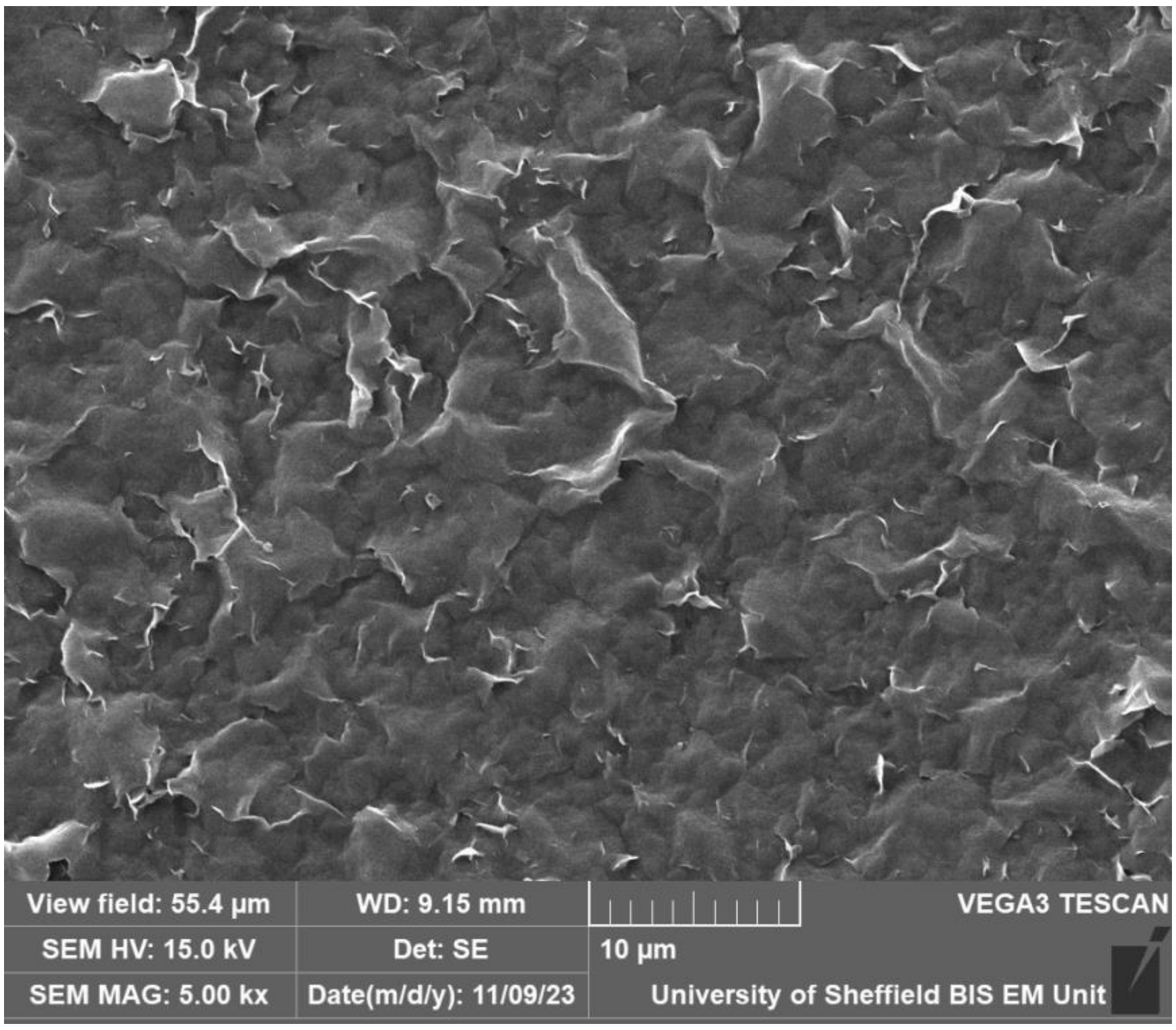

Figure S20. SEM image of Zr-TATB/PES membrane after BBG experiment.

S5. Models of Organic Dyes

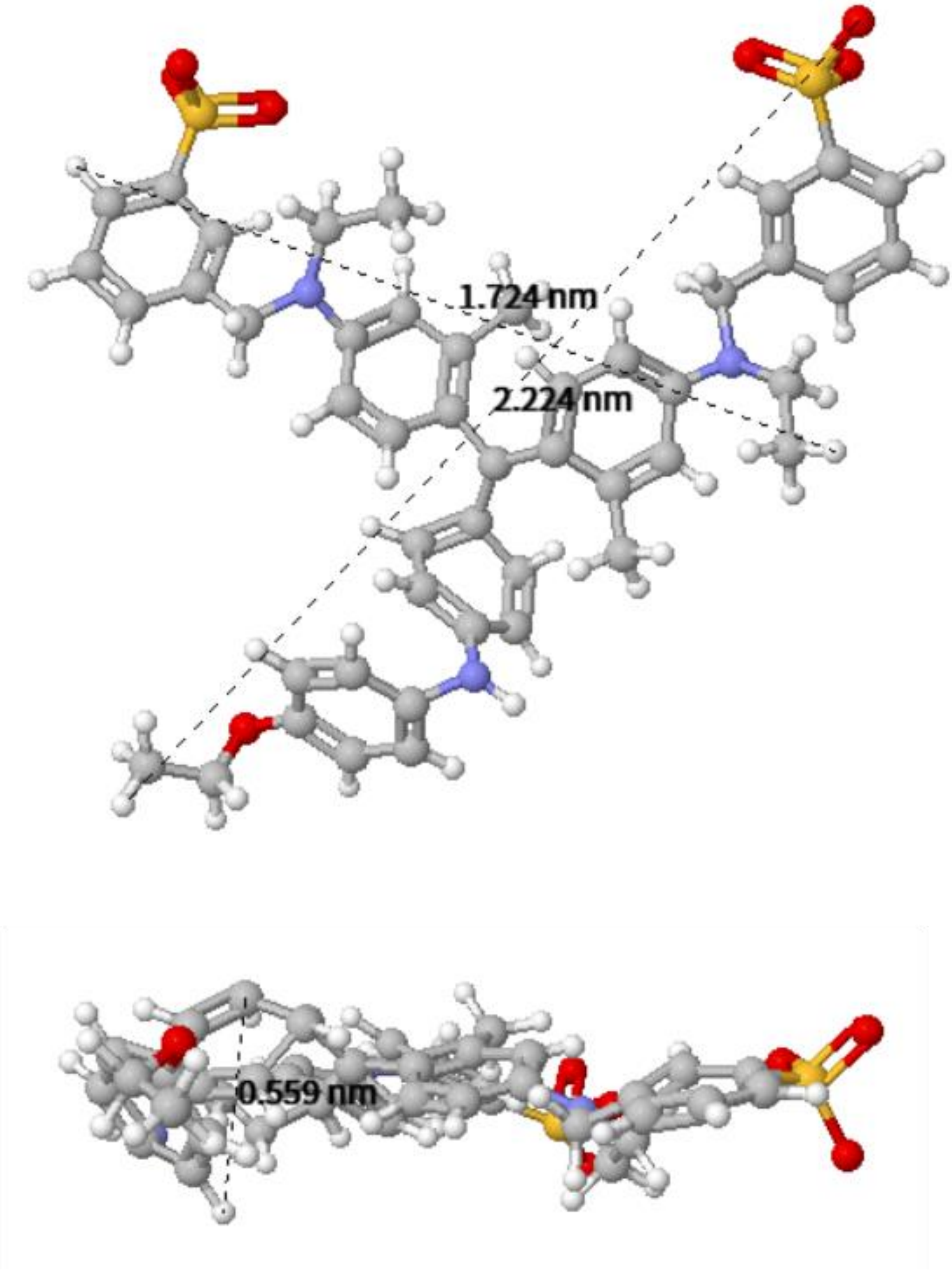

Figure S21. Model of BBG.

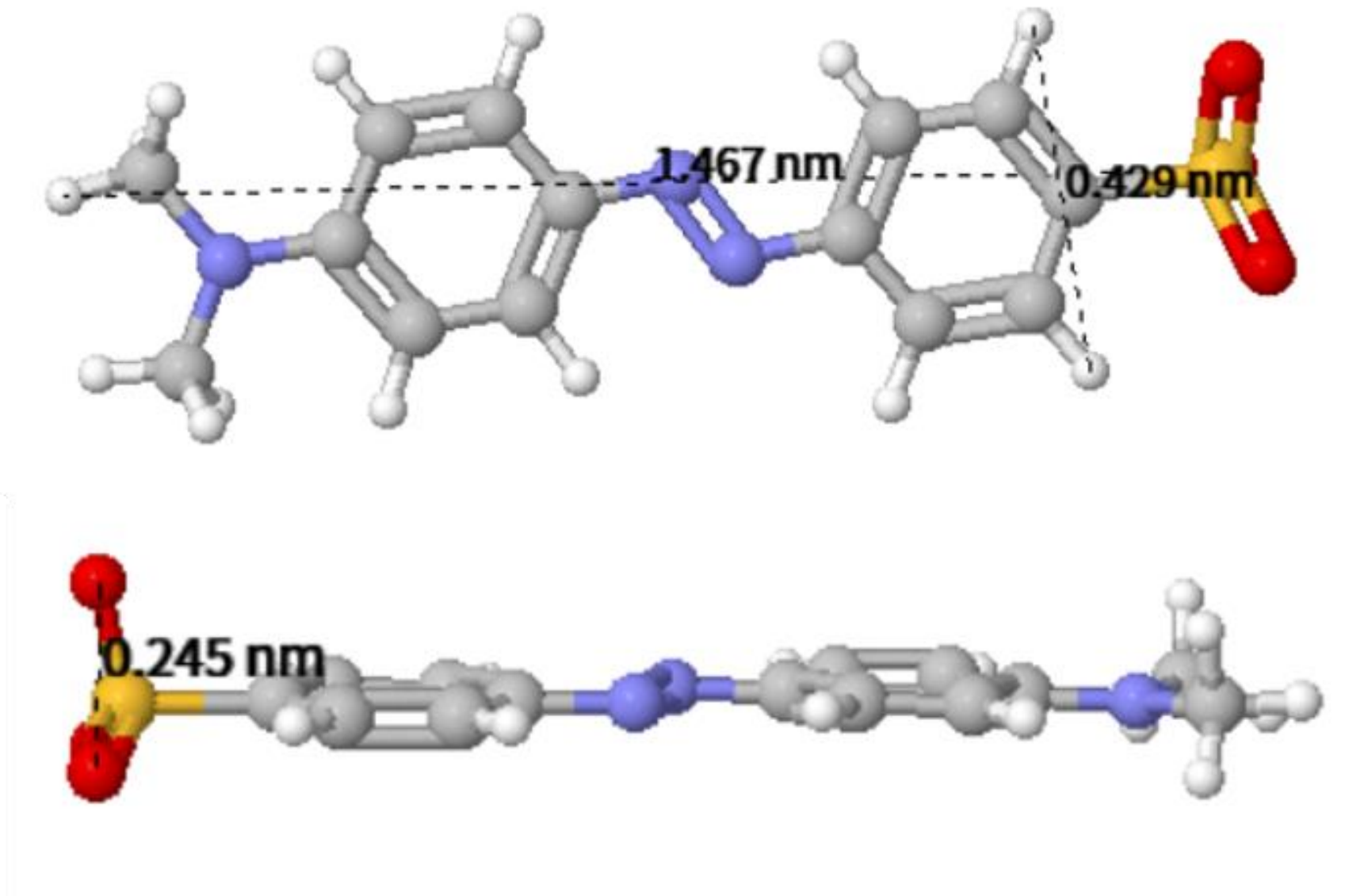

Figure S22. Model of MO.

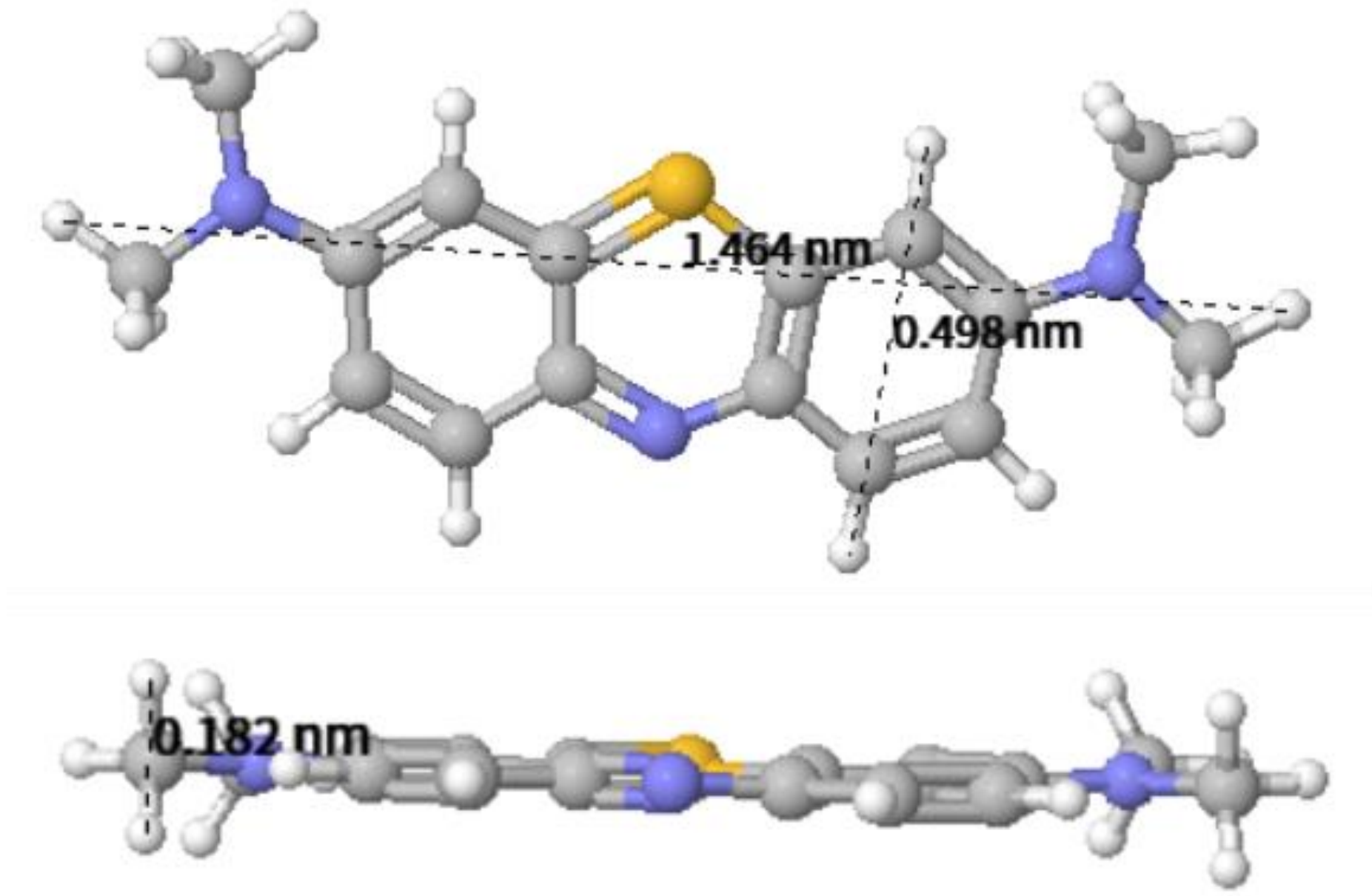

Figure S23. Model of MnB.

S6. Further studies on membranes

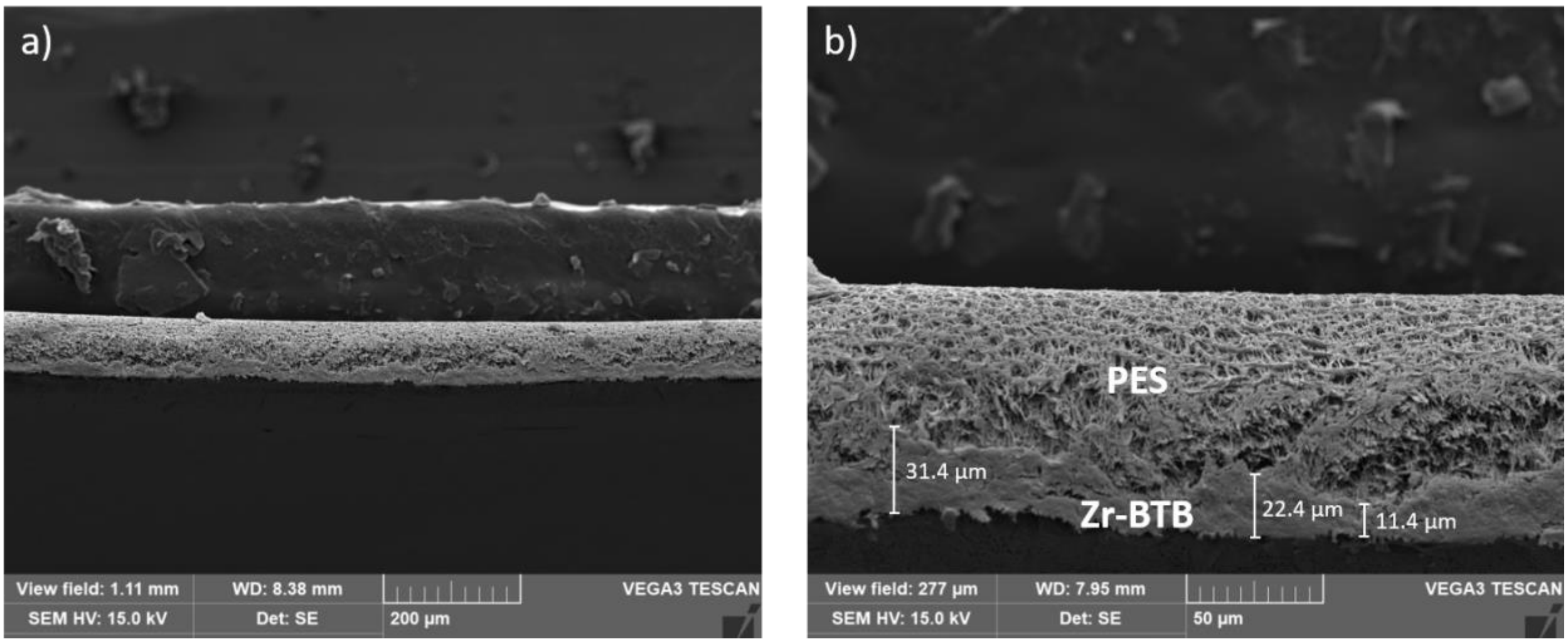

Figure S24. *Cross-sectional SEM images of Zr-BTB/PES membranes prepared by spin-coating 2.5 mg of Zr-BTB MONs onto PES supports.*

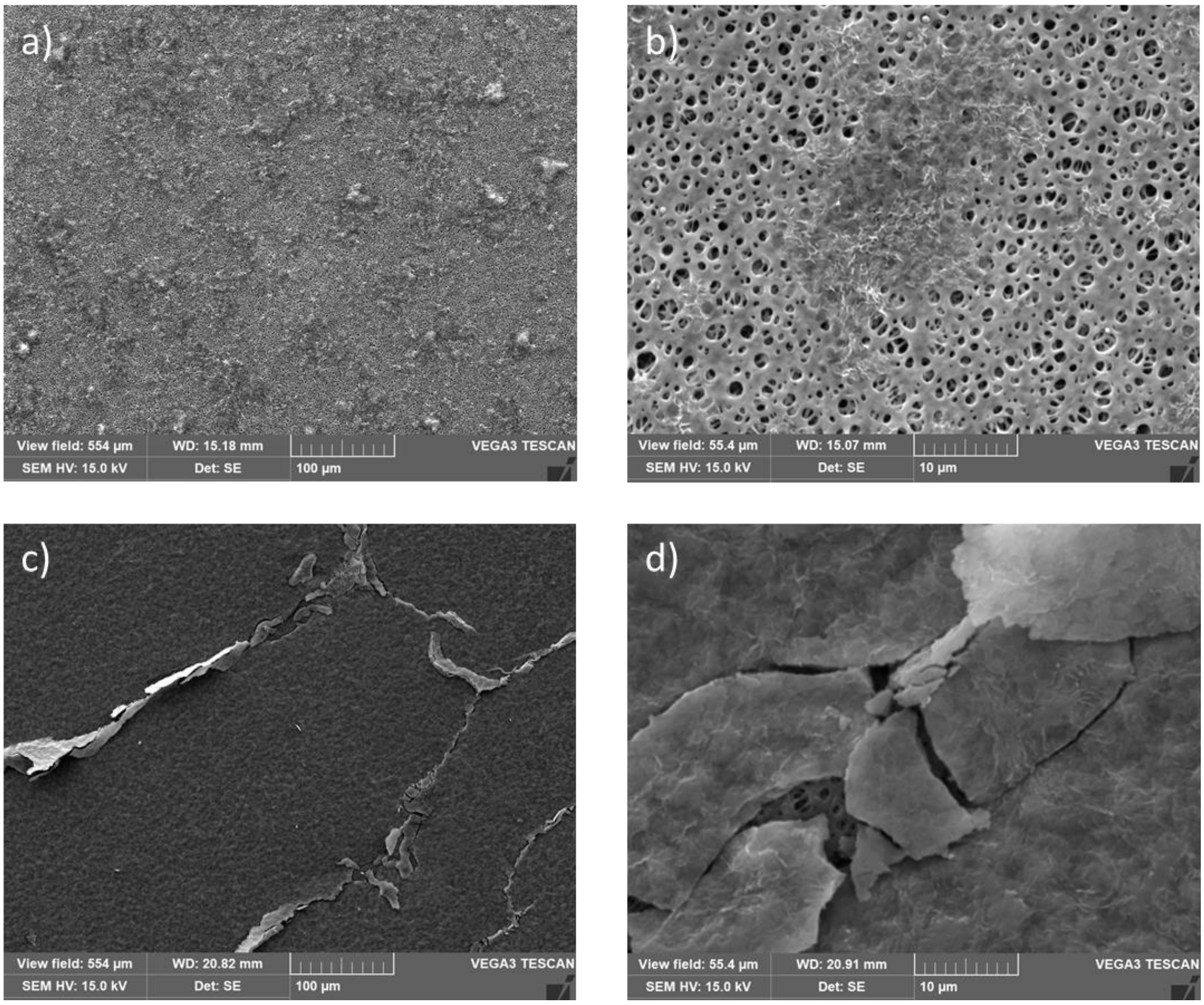

Figure S25. *SEM images of Zr-BTB/PES membranes prepared by spin-coating (a,b) 1.25 mg and (c,d) 5 mg of Zr-BTB MONs onto PES supports.*

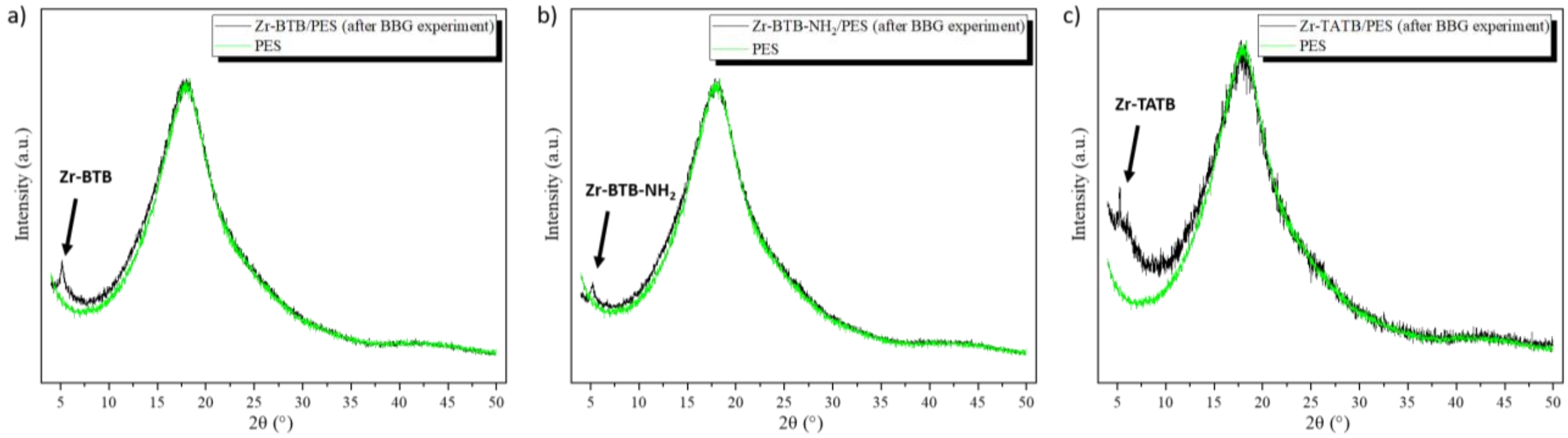

Figure S26. *PXRD characterisations of the PES support, Zr-BTB/PES, Zr-BTB-NH$_2$/PES and Zr-TATB/PES membranes after BBG experiments.*

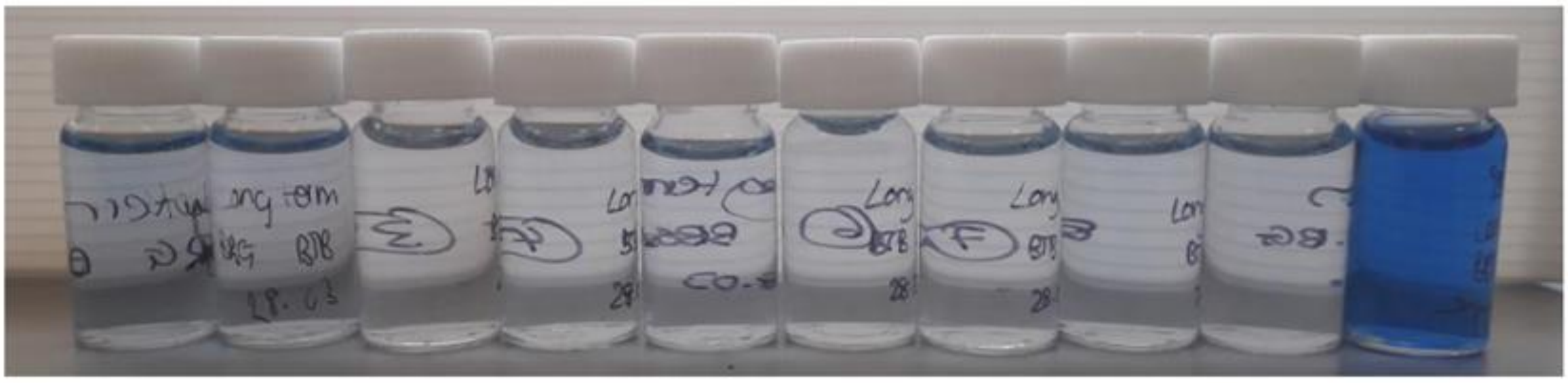

Figure S27. *Long-term stability test of Zr-BTB/PES membranes with BBG. Sequential 50 mL permeate fractions are shown from left (initial) to right (final); the far-right vial contains the initial BBG feed solution for colour comparison.*

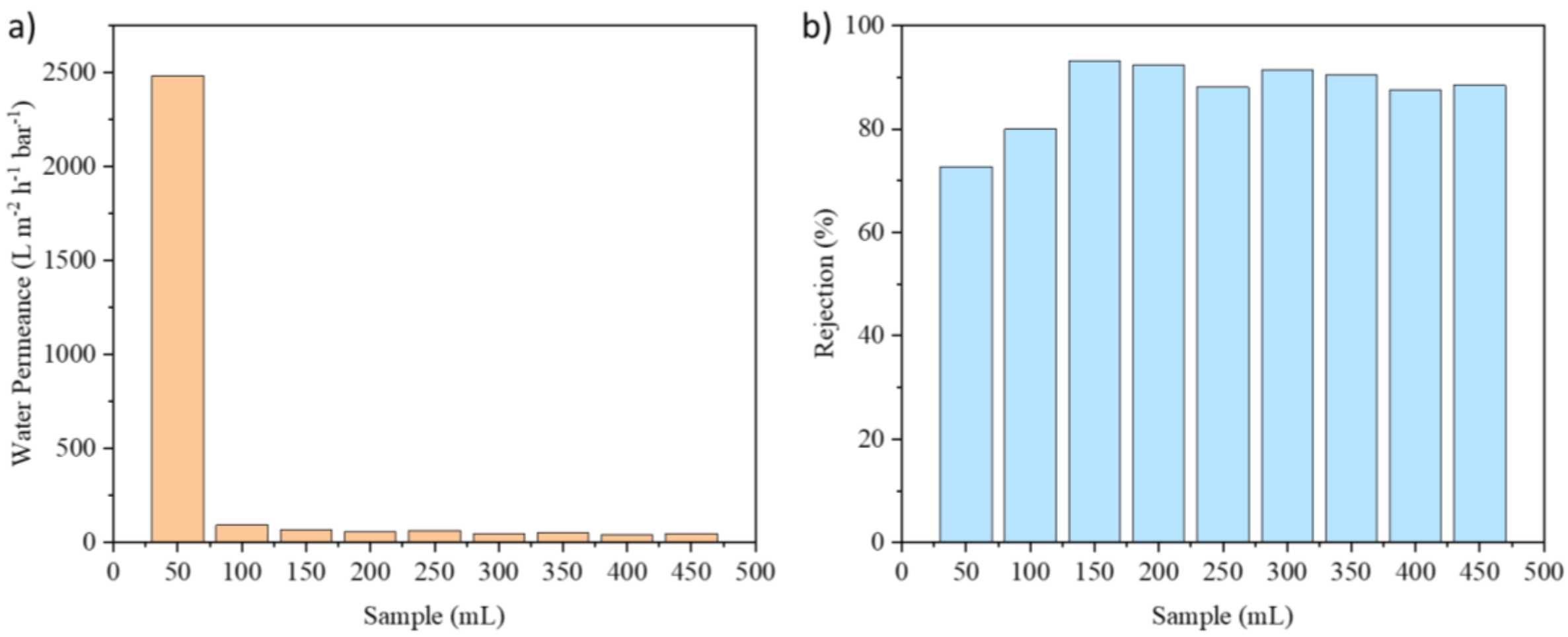

Figure S28. *Long-term stability test of Zr-BTB/PES membranes with BBG. Changes in (a) permeability and (b) rejection across 450 mL of BBG dye solution, with permeate collected in 50 mL intervals.*

Table S3. ***Dye separation performance comparison with the state-of-the-art MON-based membranes.***

Table S2. *Dye separation performance comparison with the state-of-the-art MON-based membranes.*

**Membrane**	**Dye**	**Water Permeance ($L\ m^{-2}\ h^{-1}\ bar^{-1}$)**	**Rejection (%)**	**Ref.**
Zn-TCP(Fe)-PEI/Nylon	Methyl red	4243	98.2	[3] *ACS Appl. Mater. Interfaces*, 2017
BUT-203/PEI-HPAN	Anionic dyes	87	97.9%	[4] J. *Membr. Sci.,2020*
[$Cu_2(H_4TTAC)(DMF)_2$] /Nylon	Cu phthalocyanine /phthalocyanine green	-	>99.5%	*[5] CrystEngComm , 2021*
Zr-BTB/PAN	Acid fuchsin	3.2	97.5	*[6] Adv. Mater.*, 2023
Zr-BTB-Azobenzene/PAN	Congo red<br>Acid fuchsin	15.9<br>22.7	>95<br>>95	*[7] Nat. Commun.*, 2026
Zr-BTB/PES<br>Zr-BTB-NH_2/PES<br>Zr-TATB/PES	Brilliant blue G<br>Brilliant blue G<br>Brilliant blue G	48.2 ± 26.2<br>72.9 ± 45.6<br>119.6 ± 84.3	83 ± 7<br>78.7 ± 16.3<br>72.7 ± 18.5	**This Work**

References

[1] H.-R. Fu, J. Zhang, Selective sorption of light hydrocarbons on a family of metal–organic frameworks with different imidazolate pillars, Inorg. Chem. 55 (2016) 3928–3932, https://doi.org/10.1021/acs.inorgchem.6b00136.

[2] R. R. R. Prasad. C. Pleass, A. L. Rigg, D. B. Cordes, M. M. Lozinska, V. M. Georgieva, F. Hoffmann, A. M. Z. Slawin, P. A. Wright, Isoreticular chemistry of scandium analogues of the multicomponent metal–organic framework MIL-142, CrystEngComm 23 (2021) 804-812, https://doi.org/10.1039/D0CE01593H.

[3] H. Ang, L. Hong, Polycationic polymer-regulated assembling of 2D MOF nanosheets for high-performance nanofiltration, ACS Appl. Mater. Interfaces 9 (2017) 28079–28088, https://doi.org/10.1021/acsami.7b08383.

[4] L. Shu, L.-H. Xie, Y. Meng, T. Liu, C. Zhao, J.-R. Li, A thin and high loading two-dimensional MOF nanosheet based mixed-matrix membrane for high permeance nanofiltration, J. Membr. Sci. 603 (2020) 118049, https://doi.org/10.1016/j.memsci.2020.118049.

[5] A. Li, X. Ma, J. Ma, Q. Yuan, J. Zhang, X. Xuan, A self-made portable separation device based on 2-D MOF nanosheets for the efficient separation of dyes in solutions, CrystEngComm 23 (2021) 3989–3994, https://doi.org/10.1039/d1ce00251a.

[6] H. Yuan, K. Li, D. Shi, H. Yang, X. Yu, W. Fan, P.J.S. Buenconsejo, D. Zhao, Large-area fabrication of ultrathin metal-organic framework membranes, Adv. Mater. 35 (2023) 2211859, https://doi.org/10.1002/adma.202211859.

[7] X. Peng, L. Han, X. Wu, H. L. Keok, W. Zhang, M. Mao, H. Zhu, L. Liu, H. Yang, A. Yang, J. Chu, Y. Li, S. Xiao, P. J. S. Buenconsejo, M. Zhang, D. Zhao and H. Yuan, Nat. Commun., 17, (2026), 3179, https://doi.org/10.1038/s41467-026-69929-5.
